# Supplementary material for: A 2D Gabor-wavelet baseline model out-performs a 3D surface model in scene-responsive cortex
Source: PLoS Comput Biol. 2026 Feb 2;22(2):e1013888. doi: 10.1371/journal.pcbi.1013888 (PMC12880747; doi:10.1371/journal.pcbi.1013888)
Supplement: S1 Fig — Participants’ performance was well above chance, with no obvious outliers. The difficulty of the scene task was fixed because it depended on pre-determined placement of dots on the scene. We chose these placements with the goal of keeping participants off of ceiling. We were able to staircase performance in the dot task to match the scene task, avoiding large differences in performance across runs and enabling us to determine that participants were remaining alert. (PDF) [file pcbi.1013888.s001.pdf]

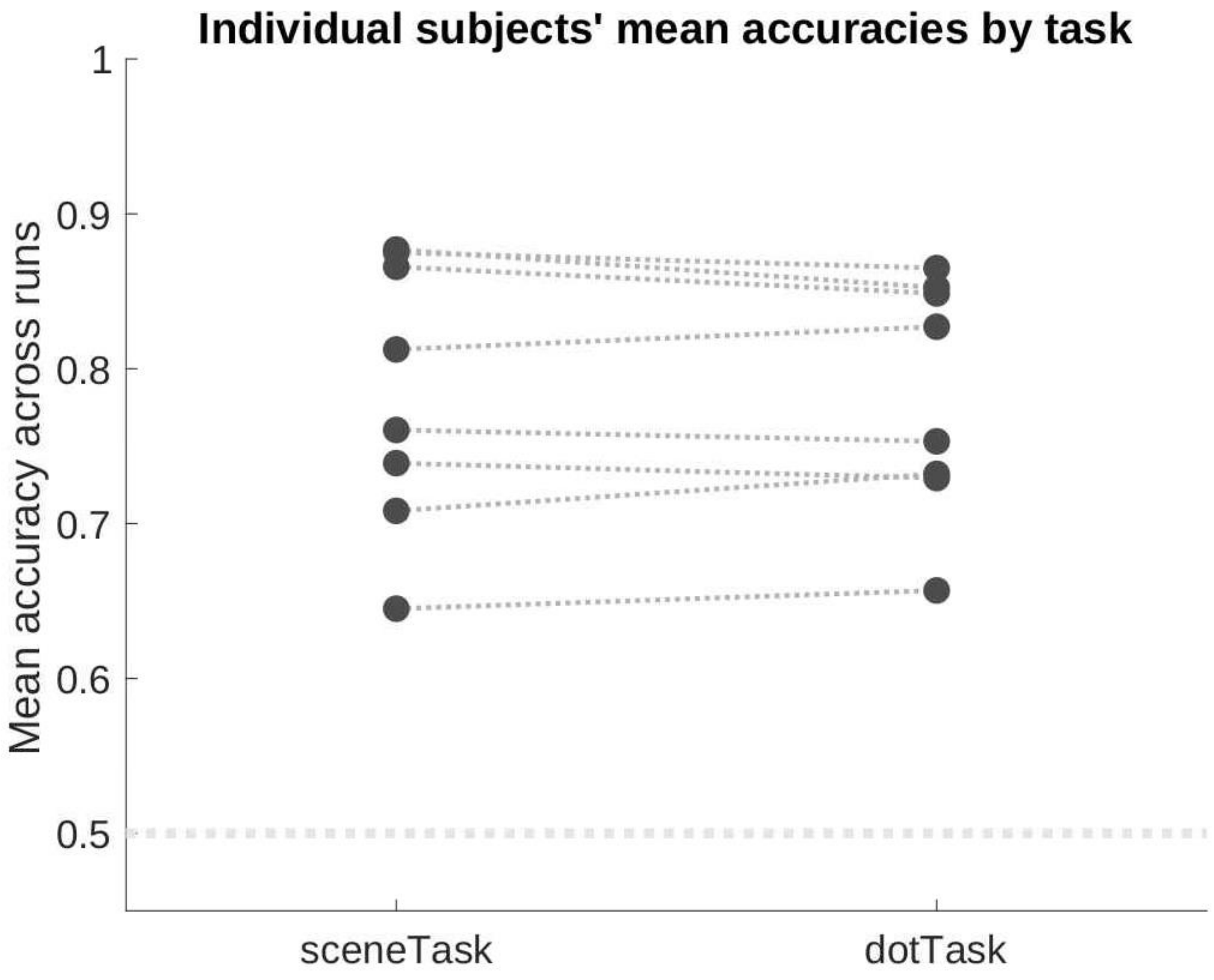

S1 Figure. Behavioral performance. Participants' performance was well above chance, with no obvious outliers. The difficulty of the scene task was fixed because it depended on pre-determined placement of dots on the scene. We chose these placements with the goal of keeping participants off of ceiling. We were able to staircase performance in the dot task to match the scene task, avoiding large differences in performance across runs and enabling us to determine that participants were remaining alert.
