# Supplementary material for: A 2D Gabor-wavelet baseline model out-performs a 3D surface model in scene-responsive cortex
Source: PLoS Comput Biol. 2026 Feb 2;22(2):e1013888. doi: 10.1371/journal.pcbi.1013888 (PMC12880747; doi:10.1371/journal.pcbi.1013888)
Supplement: S2 Fig — Results are almost identical to main results, including individual data points (cf. Fig 3, for which only V1-V3 voxels are excluded from scene-selective ROIs). (PDF) [file pcbi.1013888.s002.pdf]

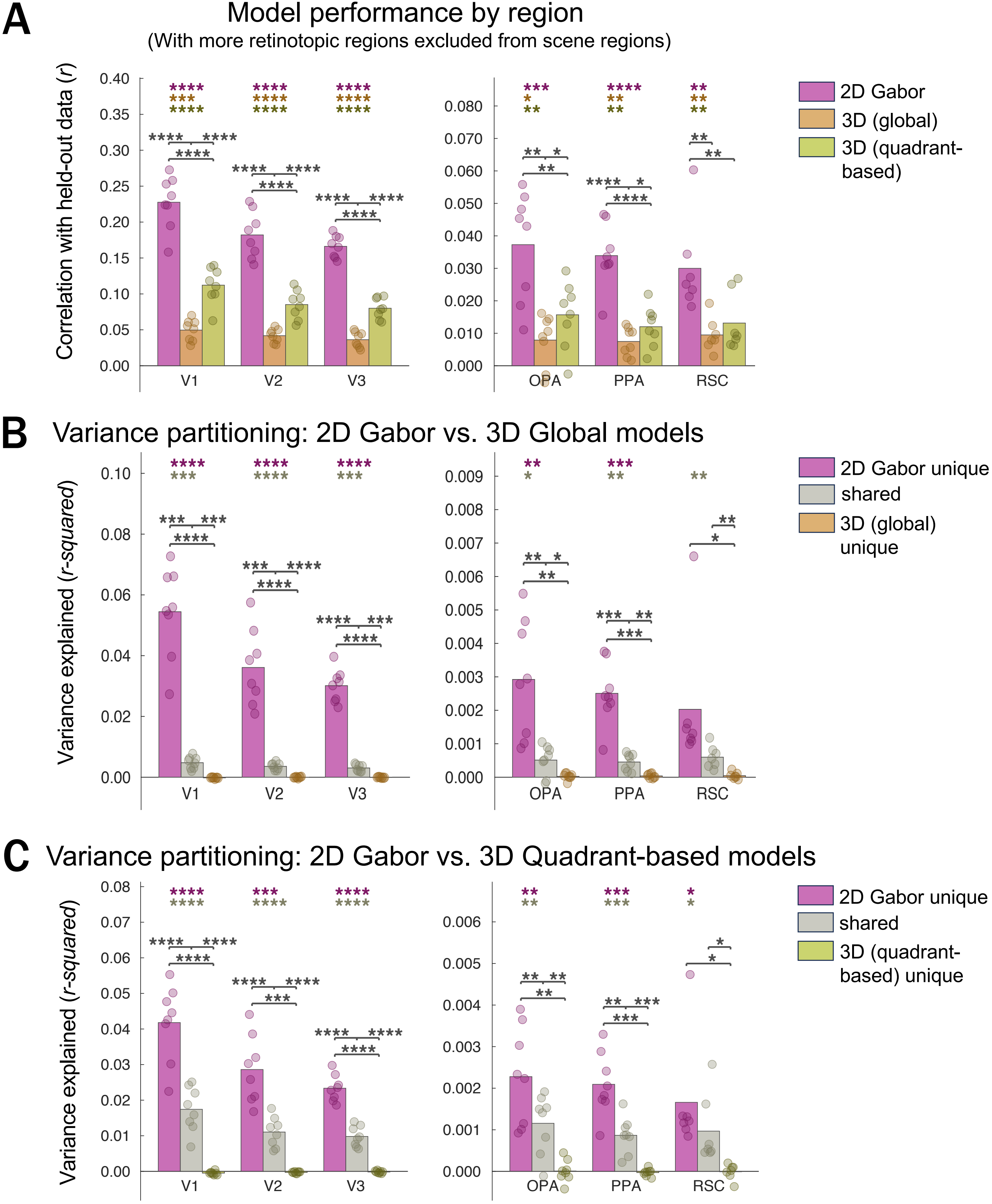
A
Model performance by region
(With more retinotopic regions excluded from scene regions)
Correlation with held-out data (r)
V1
V2
V3
OPA
PPA
RSC
2D Gabor
3D (global)
3D (quadrant-based)
B
Variance partitioning: 2D Gabor vs. 3D Global models
Variance explained (r-squared)
2D Gabor unique
shared
3D (global) unique
C
Variance partitioning: 2D Gabor vs. 3D Quadrant-based models
Variance explained (r-squared)
2D Gabor unique
shared
3D (quadrant-based) unique
