# Supplementary material for: A 2D Gabor-wavelet baseline model out-performs a 3D surface model in scene-responsive cortex
Source: PLoS Comput Biol. 2026 Feb 2;22(2):e1013888. doi: 10.1371/journal.pcbi.1013888 (PMC12880747; doi:10.1371/journal.pcbi.1013888)
Supplement: S3 Fig — Main analyses were done in volume space, and surface-space maps are for visualization purposes only. However, we have FDR-corrected significance values across all voxels with significant noise-ceiling results. (Note, however, that these analyses, like the main analyses, do not rescale model performaces by noise ceilings.) Color bar depicts the difference between the unique variance explained by the 3D global model (positive, warm colors) and the Gabor-wavelet baseline model (negative, cool colors). Panels A-G include surface maps for each of the 7 subjects not included in the main-text Fig 5 (S02-S08). (PDF) [file pcbi.1013888.s003.pdf]

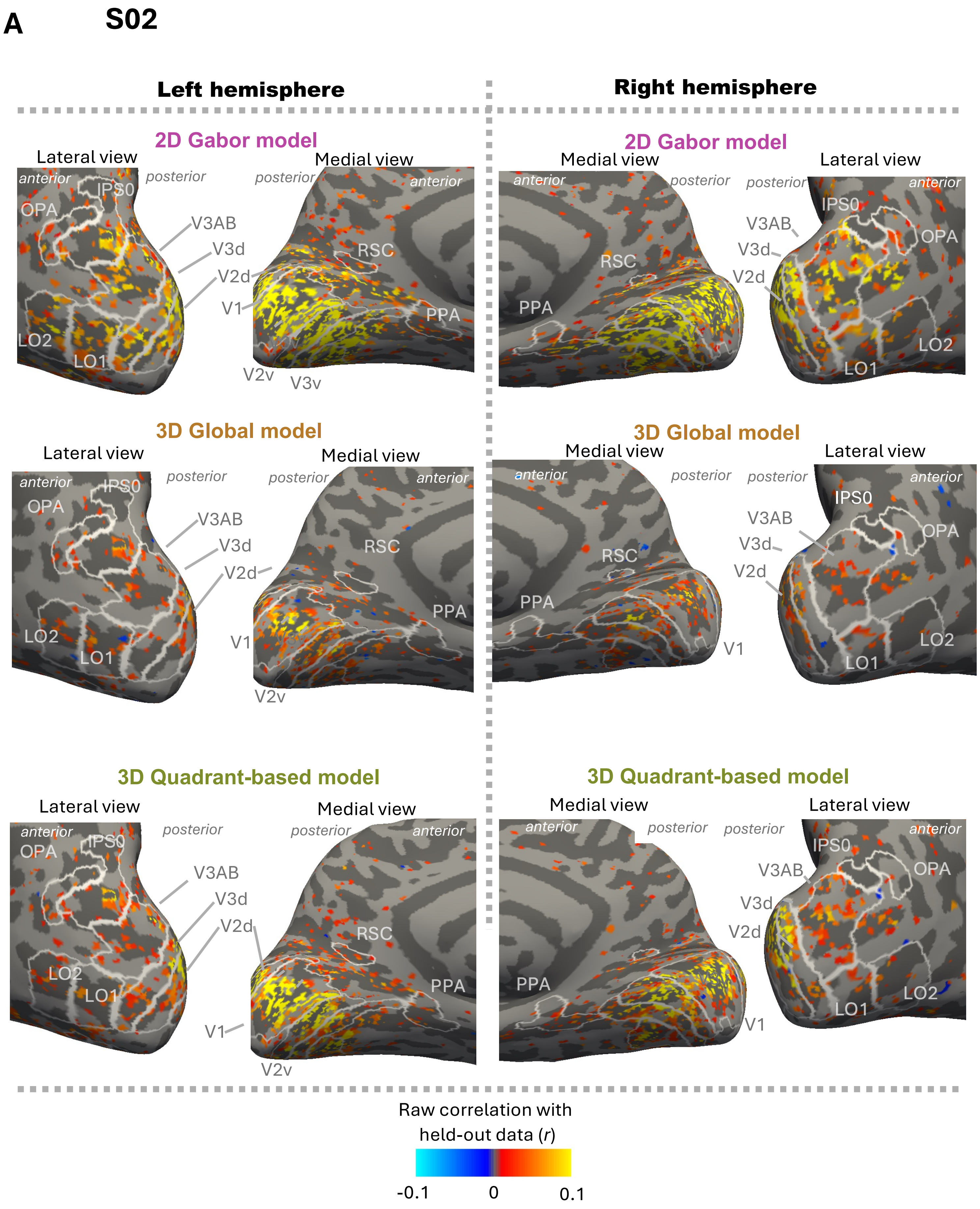
A
S02
Left hemisphere
Right hemisphere
2D Gabor model
Lateral view
Medial view
anterior
posterior
IPS0
OPA
V3AB
V3d
V2d
V1
LO2
LO1
RSC
PPA
V2v
V3v
3D Global model
3D Quadrant-based model
Raw correlation with
held-out data (r)
-0.1
0
0.1

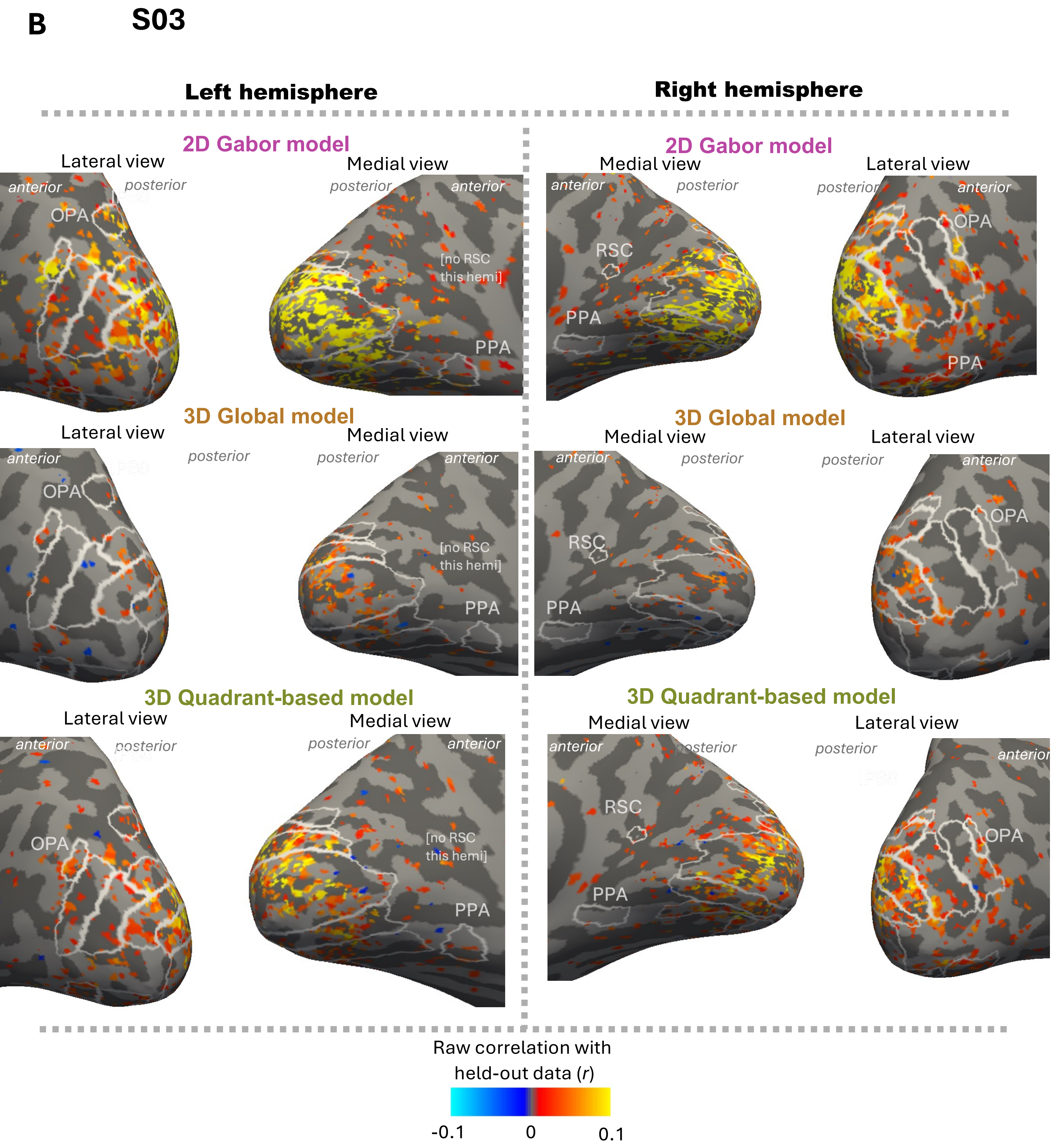
B
S03
Left hemisphere
Right hemisphere
2D Gabor model
Lateral view
Medial view
anterior
posterior
OPA
[no RSC this hemi]
PPA
RSC
3D Global model
3D Quadrant-based model
Raw correlation with held-out data (r)
-0.1
0
0.1

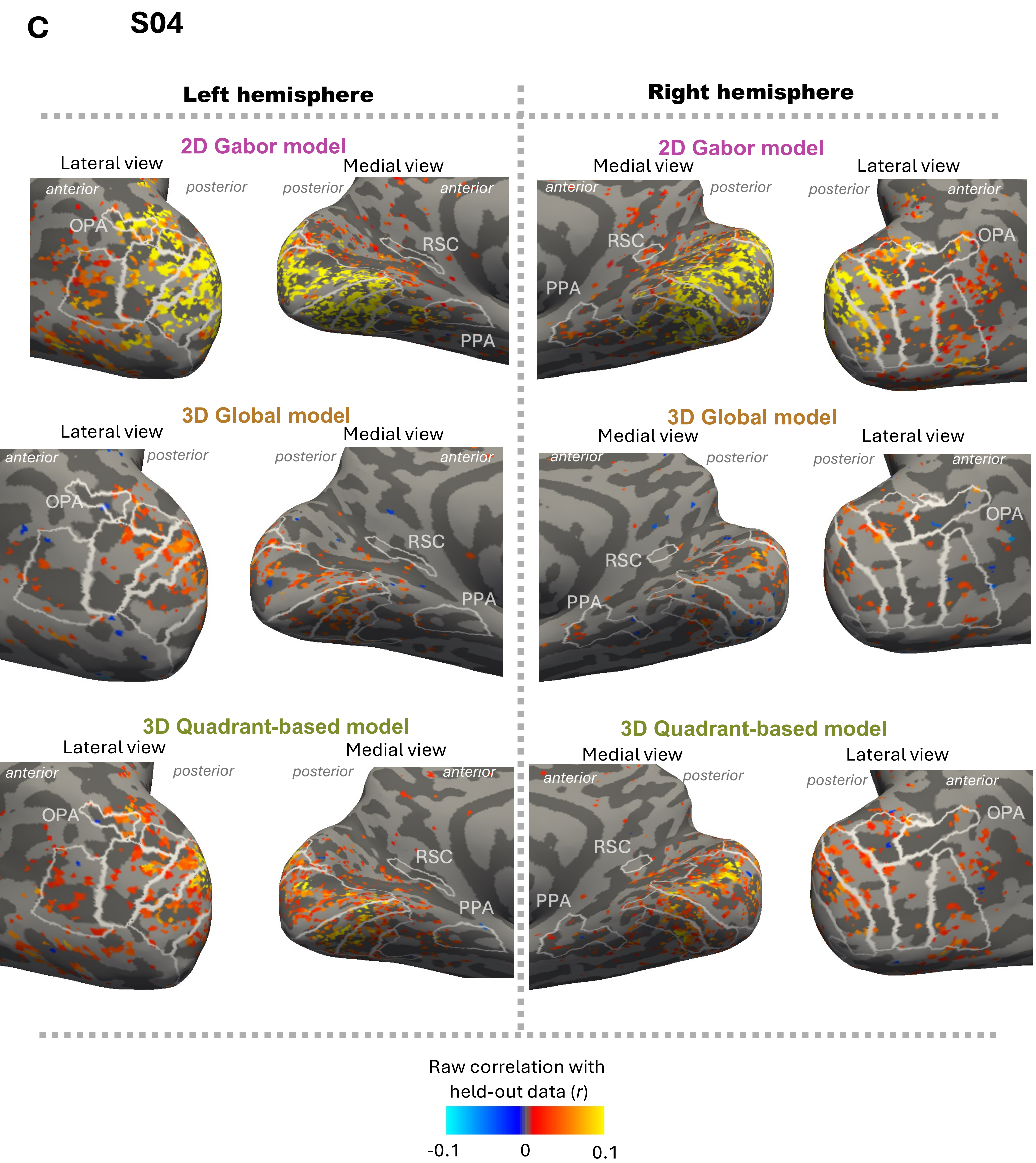
C
S04
Left hemisphere
Right hemisphere
2D Gabor model
Lateral view
Medial view
anterior
posterior
OPA
RSC
PPA
3D Global model
3D Quadrant-based model
Raw correlation with
held-out data (r)
-0.1
0
0.1

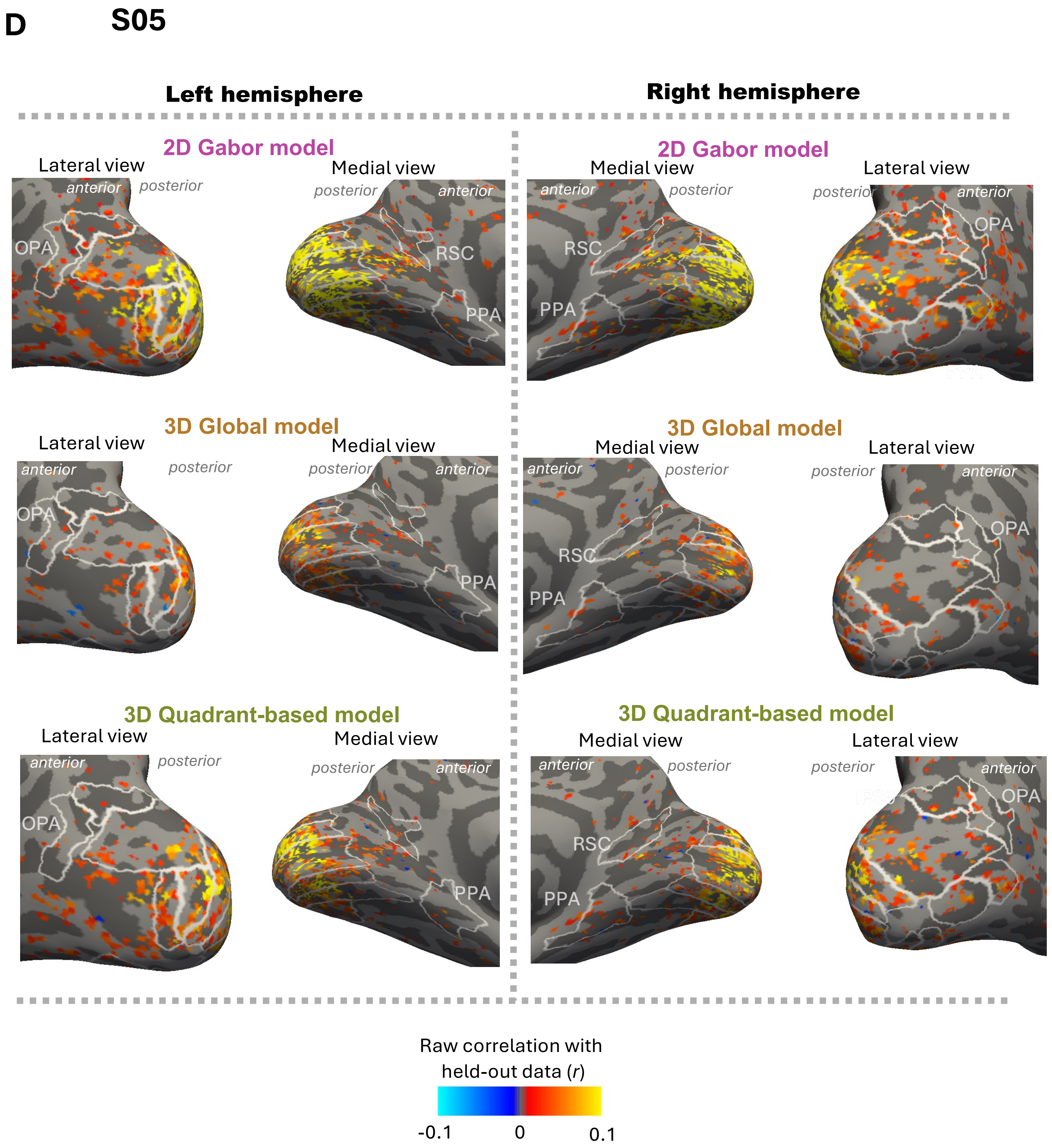
D
S05
Left hemisphere
Right hemisphere
2D Gabor model
Lateral view
Medial view
anterior
posterior
OPA
RSC
PPA
3D Global model
3D Quadrant-based model
Raw correlation with
held-out data (r)
-0.1
0
0.1

E S06

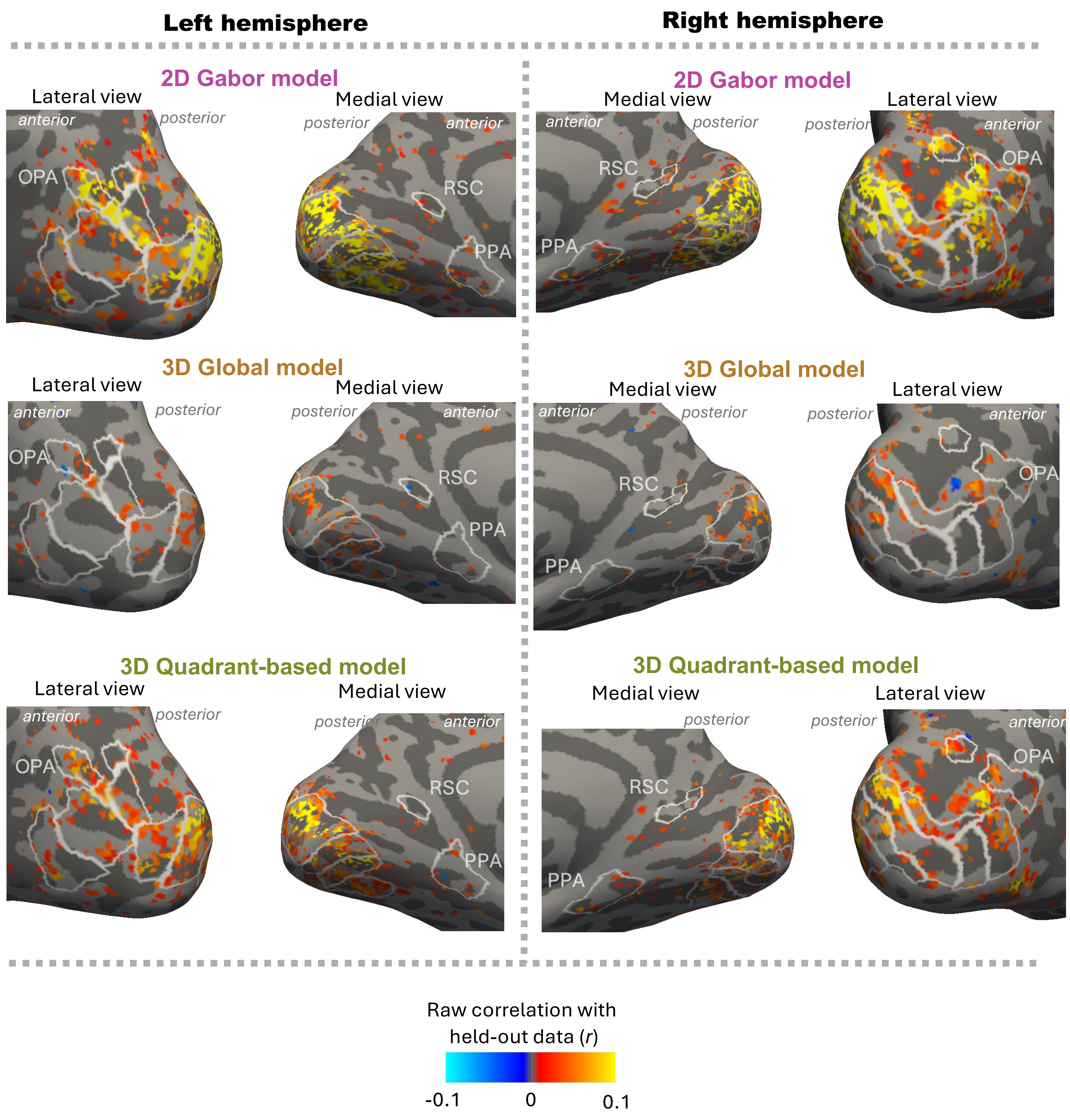

Left hemisphere
Right hemisphere
2D Gabor model
Lateral view
Medial view
anterior
posterior
OPA
RSC
PPA
3D Global model
3D Quadrant-based model
Raw correlation with held-out data (r)
-0.1
0
0.1

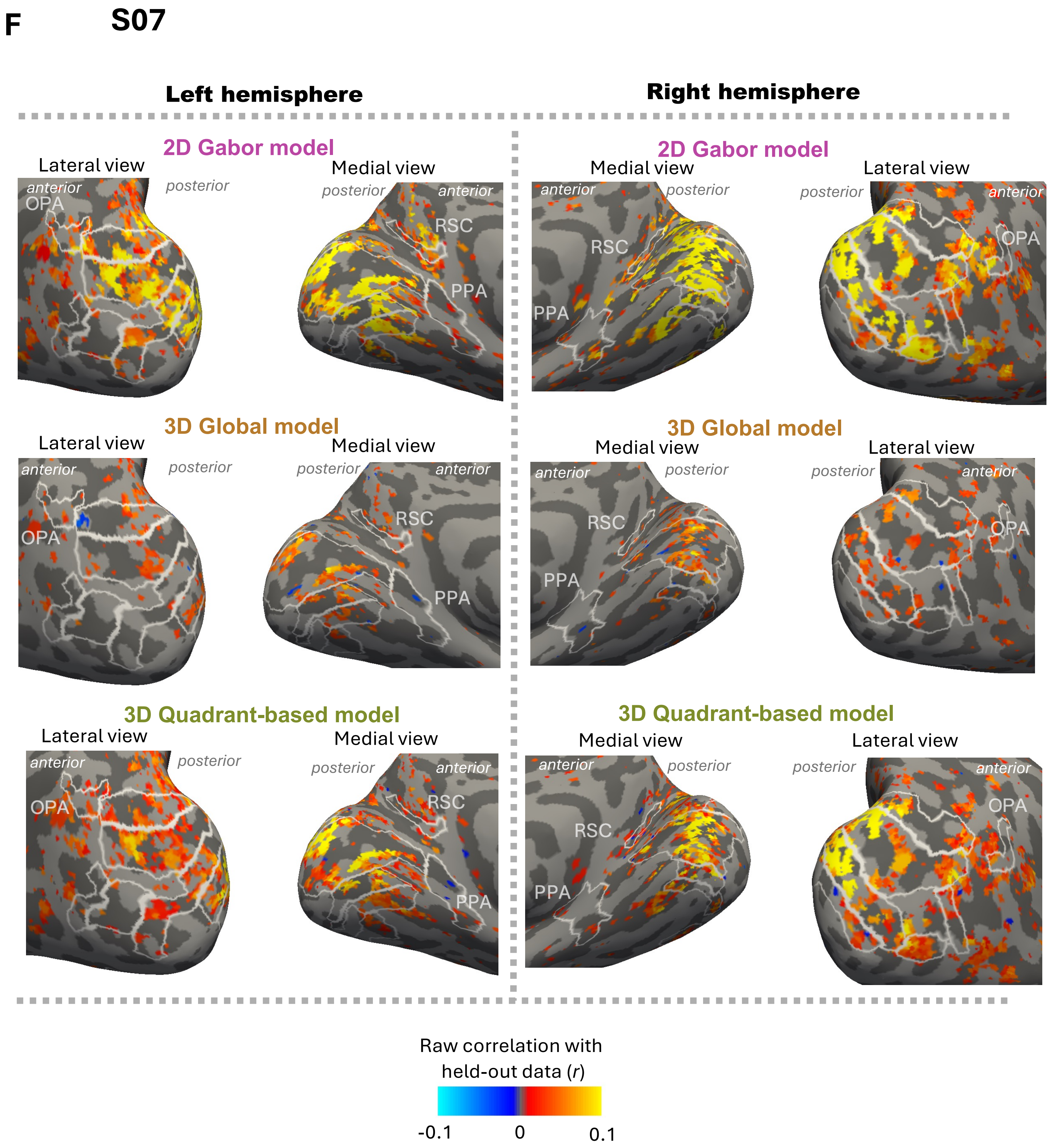
F
S07
Left hemisphere
Right hemisphere
2D Gabor model
Lateral view
Medial view
anterior
posterior
OPA
RSC
PPA
3D Global model
3D Quadrant-based model
Raw correlation with
held-out data (r)
-0.1
0
0.1

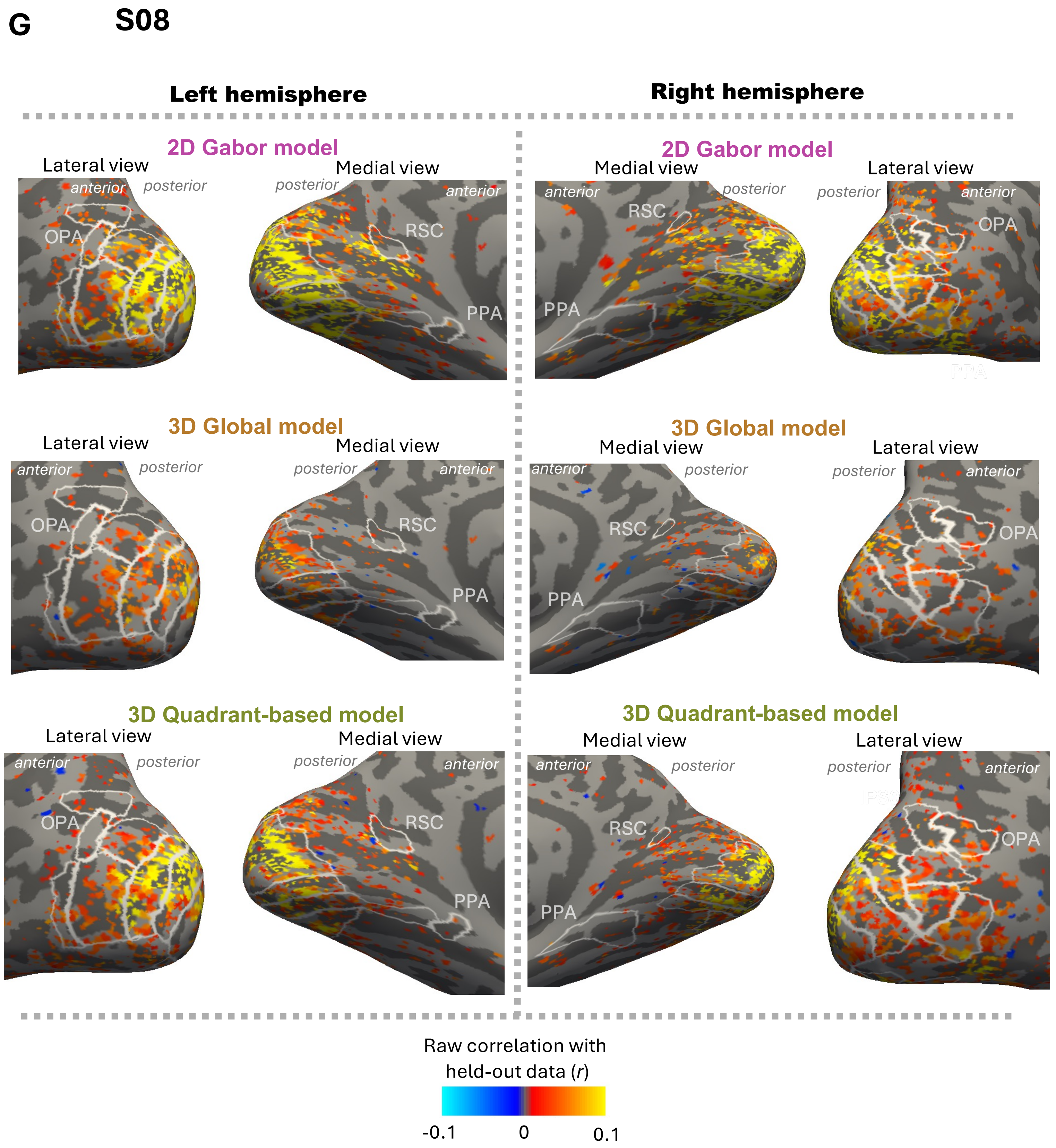
G
S08
Left hemisphere
Right hemisphere
2D Gabor model
Lateral view
Medial view
anterior
posterior
OPA
RSC
PPA
3D Global model
3D Quadrant-based model
Raw correlation with held-out data (r)
-0.1
0
0.1
