# Supplementary material for: A 2D Gabor-wavelet baseline model out-performs a 3D surface model in scene-responsive cortex
Source: PLoS Comput Biol. 2026 Feb 2;22(2):e1013888. doi: 10.1371/journal.pcbi.1013888 (PMC12880747; doi:10.1371/journal.pcbi.1013888)
Supplement: S4 Fig — Main analyses were done in volume space, and surface-space maps are for visualization purposes only. Color bar depicts the difference between the unique variance explained by the 3D global model (positive, warm colors) and the Gabor-wavelet baseline model (negative, cool colors). Panels A-G include surface maps for each of the 7 subjects not included in the main-text Fig 5 (S02-S08). (PDF) [file pcbi.1013888.s004.pdf]

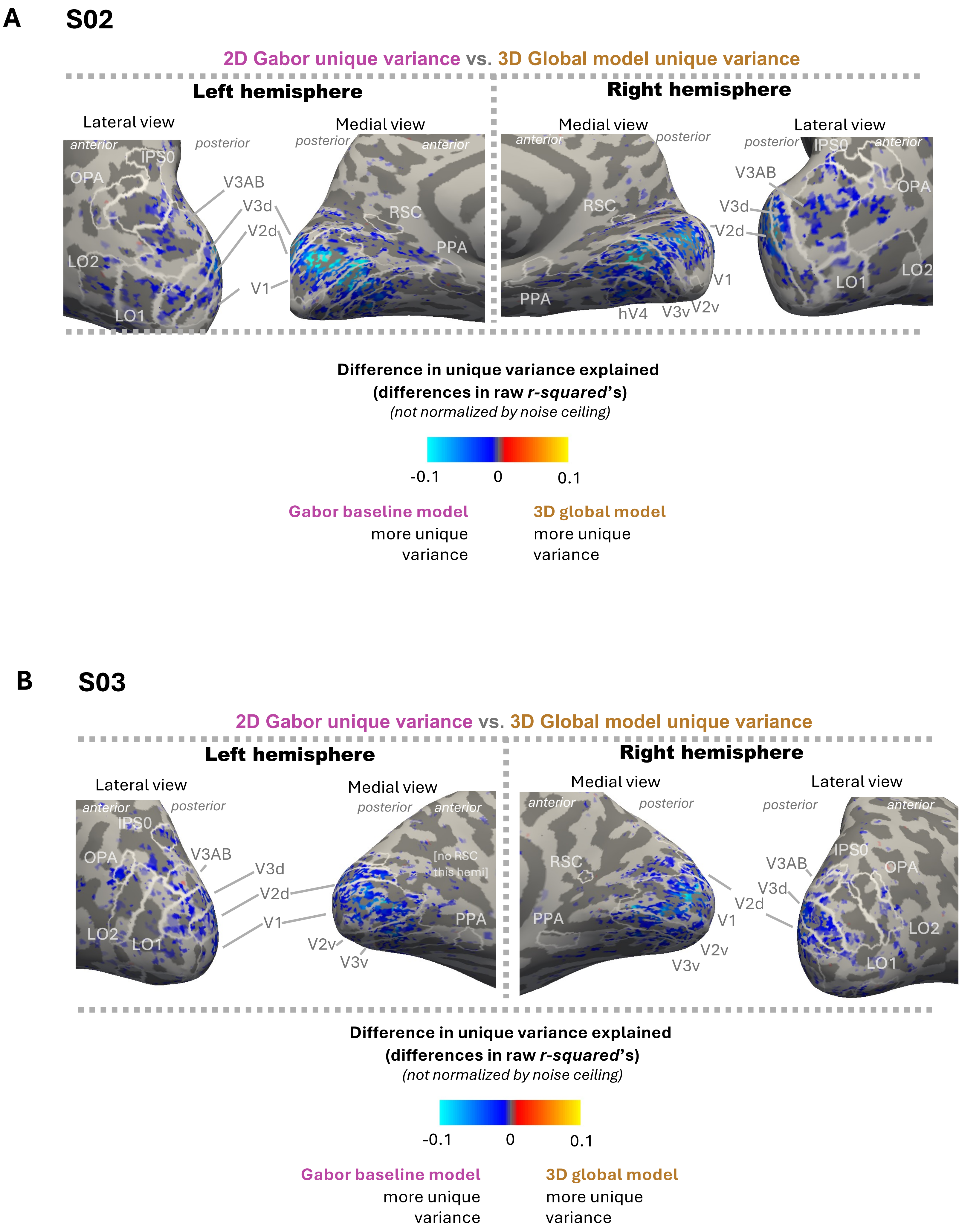
A
S02
2D Gabor unique variance vs. 3D Global model unique variance
Left hemisphere
Right hemisphere
Lateral view
Medial view
Medial view
Lateral view
anterior
posterior
posterior
anterior
anterior
posterior
posterior
anterior
IPS0
OPA
V3AB
V3d
V2d
LO2
V1
LO1
RSC
PPA
RSC
PPA
hV4
V3v
V2v
V1
V3AB
V3d
V2d
IPS0
OPA
LO1
LO2
Difference in unique variance explained
(differences in raw r-squared's)
(not normalized by noise ceiling)
-0.1
0
0.1
Gabor baseline model
more unique
variance
3D global model
more unique
variance
B
S03
2D Gabor unique variance vs. 3D Global model unique variance
Left hemisphere
Right hemisphere
Lateral view
Medial view
Medial view
Lateral view
anterior
posterior
posterior
anterior
anterior
posterior
posterior
anterior
IPS0
OPA
V3AB
V3d
V2d
V1
LO2
LO1
[no RSC
this hemi]
PPA
V2v
V3v
RSC
PPA
V2d
V1
V2v
V3v
V3AB
V3d
IPS0
OPA
LO2
LO1
Difference in unique variance explained
(differences in raw r-squared's)
(not normalized by noise ceiling)
-0.1
0
0.1
Gabor baseline model
more unique
variance
3D global model
more unique
variance

C **S04**

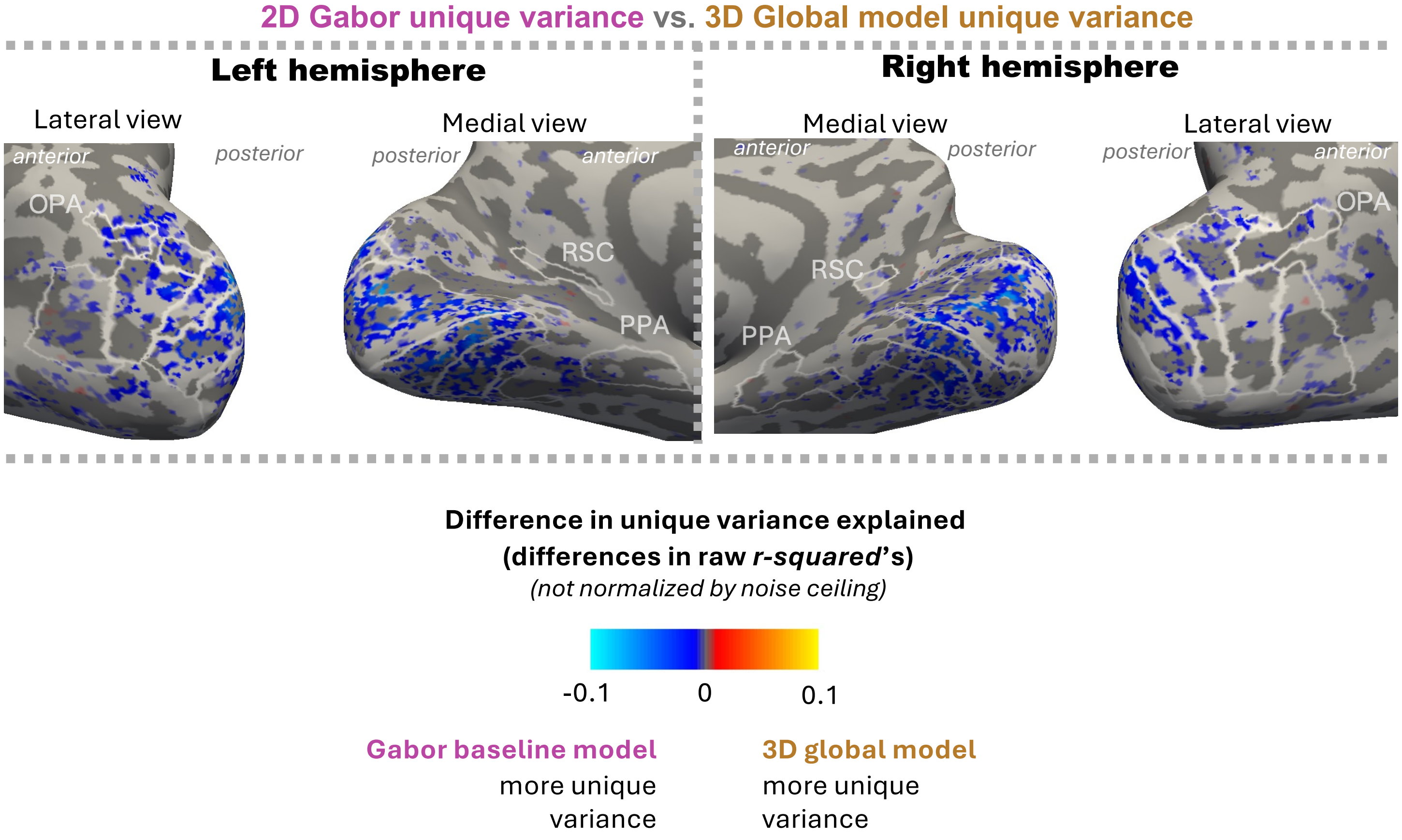
2D Gabor unique variance vs. 3D Global model unique variance
Left hemisphere
Right hemisphere
Lateral view
Medial view
Medial view
Lateral view
anterior
posterior
posterior
anterior
anterior
posterior
posterior
anterior
OPA
RSC
PPA
RSC
PPA
OPA
Difference in unique variance explained
(differences in raw r-squared's)
(not normalized by noise ceiling)
-0.1
0
0.1
Gabor baseline model
more unique
variance
3D global model
more unique
variance

D **S05**

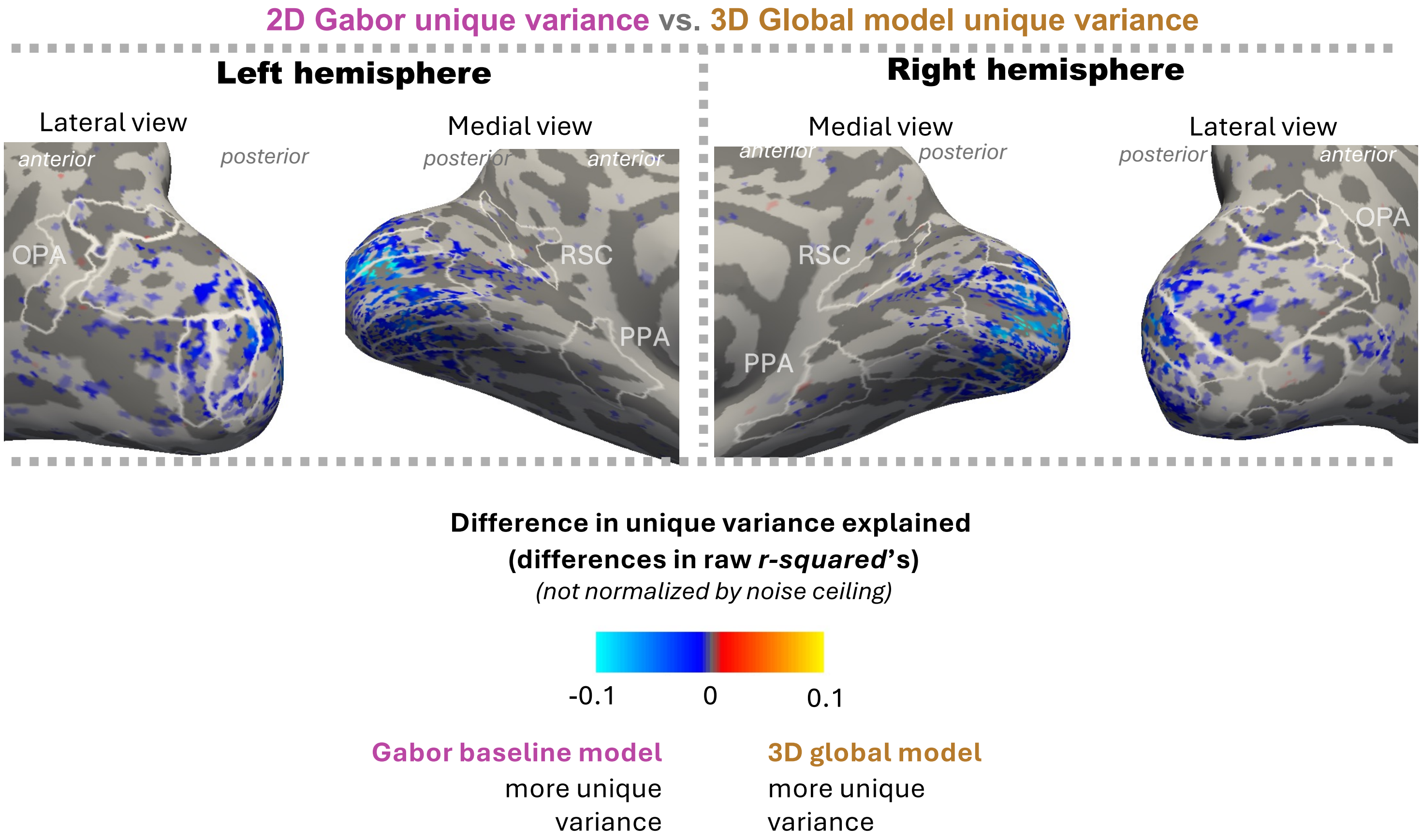
2D Gabor unique variance vs. 3D Global model unique variance
Left hemisphere
Right hemisphere
Lateral view
Medial view
Medial view
Lateral view
anterior
posterior
posterior
anterior
anterior
posterior
posterior
anterior
OPA
RSC
PPA
RSC
PPA
OPA
Difference in unique variance explained
(differences in raw r-squared's)
(not normalized by noise ceiling)
-0.1
0
0.1
Gabor baseline model
more unique
variance
3D global model
more unique
variance

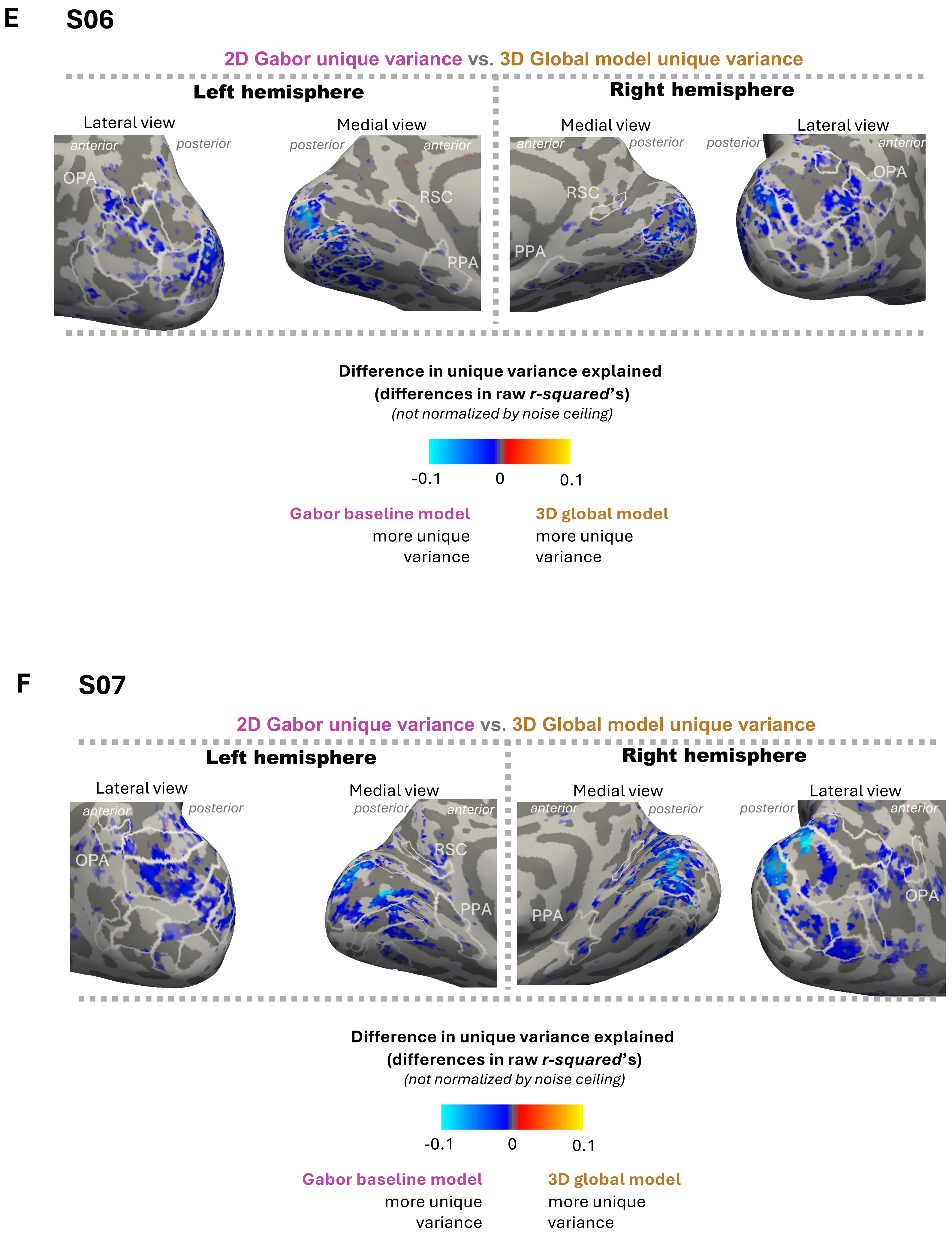
E
S06
2D Gabor unique variance vs. 3D Global model unique variance
Left hemisphere
Right hemisphere
Lateral view
Medial view
Medial view
Lateral view
anterior
posterior
posterior
anterior
anterior
posterior
posterior
anterior
OPA
RSC
PPA
RSC
PPA
OPA
Difference in unique variance explained
(differences in raw r-squared's)
(not normalized by noise ceiling)
-0.1
0
0.1
Gabor baseline model
more unique
variance
3D global model
more unique
variance
F
S07
2D Gabor unique variance vs. 3D Global model unique variance
Left hemisphere
Right hemisphere
Lateral view
Medial view
Medial view
Lateral view
anterior
posterior
posterior
anterior
anterior
posterior
posterior
anterior
OPA
RSC
PPA
PPA
OPA
Difference in unique variance explained
(differences in raw r-squared's)
(not normalized by noise ceiling)
-0.1
0
0.1
Gabor baseline model
more unique
variance
3D global model
more unique
variance

G **S08**

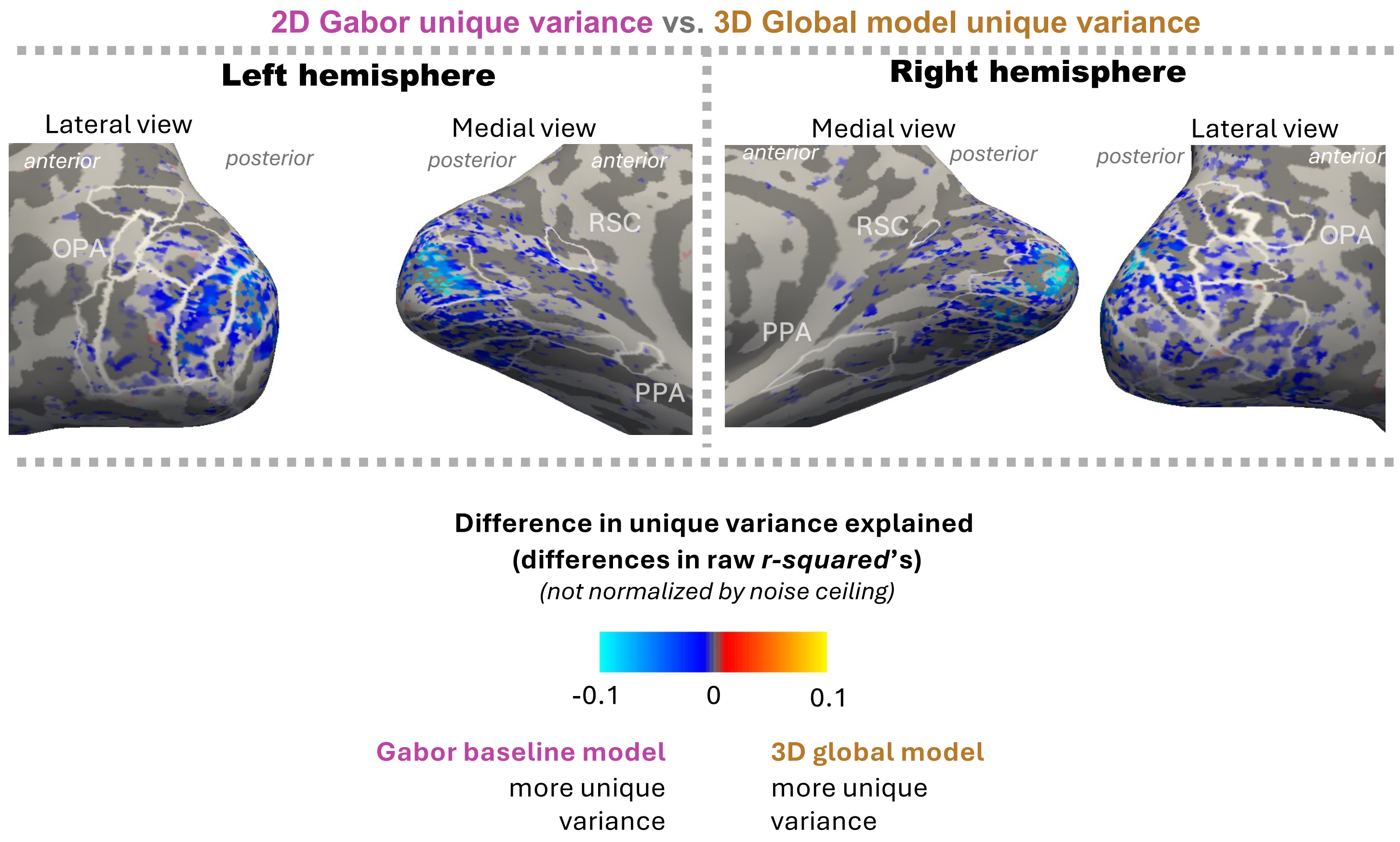
2D Gabor unique variance vs. 3D Global model unique variance
Left hemisphere
Right hemisphere
Lateral view
Medial view
Medial view
Lateral view
anterior
posterior
posterior
anterior
anterior
posterior
posterior
anterior
OPA
RSC
PPA
RSC
PPA
OPA
Difference in unique variance explained
(differences in raw r-squared's)
(not normalized by noise ceiling)
-0.1
0
0.1
Gabor baseline model
more unique
variance
3D global model
more unique
variance
