# Supplementary material for: A 2D Gabor-wavelet baseline model out-performs a 3D surface model in scene-responsive cortex
Source: PLoS Comput Biol. 2026 Feb 2;22(2):e1013888. doi: 10.1371/journal.pcbi.1013888 (PMC12880747; doi:10.1371/journal.pcbi.1013888)
Supplement: S1 Table — For the present stimuli, only the 3D-global scene-surface model was included, as it most closely corresponded to Lescroart & Gallant’s (2019) model [13]. (PDF) [file pcbi.1013888.s005.pdf]

S1 Table. RDM-correlations between 2D gabor-wavelet model and 3D-global scene-surface models. Correlations are similar within stimulus sets across 3 distance metrics. For the present stimuli, only the 3D-global scene-surface model was included, as it most closely corresponded to Lescroart & Gallant's (2019) model [13].

**Distance metric**	**Present stimuli**	**Lescroart & Gallant (2019) *[13]***
Cosine distance	-0.002	0.274
Spearman's rho	-0.001	0.191
Pearson correlation	-0.003	0.277
