# Supplementary material for: A 2D Gabor-wavelet baseline model out-performs a 3D surface model in scene-responsive cortex
Source: PLoS Comput Biol. 2026 Feb 2;22(2):e1013888. doi: 10.1371/journal.pcbi.1013888 (PMC12880747; doi:10.1371/journal.pcbi.1013888)
Supplement: S2 Table — A) Two-tailed p-values corresponding to permutation tests for each model compared to 0, as well as comparisons of performance for each. B) Variance partitioning, two-tailed p-values corresponding to permutation tests for 3Dquadrant-based and Gabor models. C) Variance partitioning, two-tailed p-values corresponding to permutation tests for 3D-global and Gabor models. (PDF) [file pcbi.1013888.s006.pdf]

S2 Table. Statistical tests for main analyses in text.

S2 Table A): Two-tailed p-values corresponding to permutation tests for each model compared to 0, as well as comparisons of performance for each.

ROI	Gabor-wavelet baseline ("2D") model	3D global model	3D Quadrant-based model
V1	0.0001	0.0001	0.0001
V2	0.0001	0.0001	0.0002
V3	0.0001	0.0001	0.0001
OPA	0.0002	0.0132	0.0009
PPA	0.0001	0.0019	0.0016
RSC	0.0018	0.0040	0.0099

ROI	"2D" Gabor vs. 3D Global	"2D" Gabor vs. 3D Quadrant-based	3D Global vs. 3D Quadrant-based
V1	0.0001	0.0001	0.0001
V2	0.0001	0.0001	0.0002
V3	0.0001	0.0001	0.0001
OPA	0.0008	0.0002	0.0342
PPA	0.0001	0.0001	0.0221
RSC	0.0025	0.0005	0.1638

S2 Table B): Variance partitioning, two-tailed p-values corresponding to permutation tests for 3D-quadrant-based and Gabor models.

ROI	Unique Gabor-wavelet ("2D") variance	Shared variance	Unique 3D-quadrant-based variance
V1	0.0001	0.0002	0.0261
V2	0.0002	0.0002	0.0088
V3	0.0001	0.0001	0.1662
OPA	0.0006	0.0019	0.6623
PPA	0.0002	0.0004	0.4103
RSC	0.0257	0.0259	0.8987

ROI	Unique Gabor variance vs. shared variance	Unique Gabor variance vs. unique 3D-quadrant-based variance	Unique 3D-quadrant-based variance vs. shared variance
V1	0.0001	0.0001	0.0001
V2	0.0001	0.0002	0.0002
V3	0.0002	0.0001	0.0001
OPA	0.0027	0.0008	0.0020
PPA	0.0011	0.0002	0.0002
RSC	0.0367	0.0155	0.0193

S2 Table C): Variance partitioning, two-tailed p-values corresponding to permutation tests for 3D-global and Gabor models.

ROI	Unique Gabor-wavelet (“2D”) variance	Shared variance	Unique 3D-global variance
V1	0.0001	0.0007	0.0262
V2	0.0001	0.0001	0.7131
V3	0.0001	0.0003	0.3412
OPA	0.0018	0.0069	0.2607
PPA	0.0005	0.0018	0.1288
RSC	0.0526	0.0048	0.2874

ROI	Unique Gabor variance vs. shared variance	Unique Gabor variance vs. Unique 3D-global variance	Unique 3D-global variance vs. shared variance
V1	0.0001	0.0001	0.0007
V2	0.0002	0.0001	0.0001
V3	0.0001	0.0001	0.0002
OPA	0.0061	0.0016	0.0040
PPA	0.0002	0.0003	0.0015
RSC	0.0793	0.0297	0.0010
