# Supplementary material for: A 2D Gabor-wavelet baseline model out-performs a 3D surface model in scene-responsive cortex
Source: PLoS Comput Biol. 2026 Feb 2;22(2):e1013888. doi: 10.1371/journal.pcbi.1013888 (PMC12880747; doi:10.1371/journal.pcbi.1013888)
Supplement: S3 Table — ANOVA contains main effects of ROI and Condition (Model), as well as the interaction between them. Not only do we find extremely reliable main effects of ROI and model, but critically we also find an extremely significant interaction between the two. While the Gabor model is the strongest model in every region, this interaction reflects the magnitude of the Gabor advantage decreasing from early visual regions to scene regions (Fig 3A). (PDF) [file pcbi.1013888.s007.pdf]

S3 Table: A repeated-measures ANOVA on model performances across ROIs, containing main effects of ROI and Condition (Model), as well as the interaction between them. Not only do we find extremely reliable main effects of ROI and model, but critically we also find an extremely significant interaction between the two. While the Gabor model is the strongest model in every region, this interaction reflects the magnitude of the *Gabor advantage* decreasing from early visual regions to scene regions (Figure 3A).

	F Value	Num. DF	Den. DF	P-value
ROI	99.644375	5.0	30.0	9.363692e-18
Condition (Model)	749.430270	2.0	12.0	2.510390e-13
ROI x Condition intxn.	99.208901	10.0	60.0	1.780918e-33
