# Supplementary material for: A 2D Gabor-wavelet baseline model out-performs a 3D surface model in scene-responsive cortex
Source: PLoS Comput Biol. 2026 Feb 2;22(2):e1013888. doi: 10.1371/journal.pcbi.1013888 (PMC12880747; doi:10.1371/journal.pcbi.1013888)
Supplement: Attachment — Submitted filename: response_to_reviewers_clean.pdf [file pcbi.1013888.s010.pdf]

UNIVERSITY OF CALIFORNIA SAN DIEGO

BERKELEY • DAVIS • IRVINE • LOS ANGELES • MERCED • RIVERSIDE • SAN DIEGO • SAN FRANCISCO SANTA BARBARA • SANTA CRUZ

September 3, 2024

Dear Dr. Kietzmann and Dr. Martin,

Thank you for your feedback on our manuscript "A 2D Gabor-wavelet baseline model out-performs a 3D surface model in scene-responsive cortex" (PCOMPBIOL-D-24-00406). We greatly appreciate the effort of yourself and reviewers, and we believe the paper is significantly improved because of this feedback. In our point-by-point response below, we highlight the changes we made in our manuscript to address the concerns of the reviewers. In our revised manuscript, we have highlighted the changed sections in **orange** for clarity.

We hope that the present revision may be viewed as a substantial improvement that will be acceptable for publication. Please don't hesitate to contact us if you have any further questions, and we look forward to hearing from you. Thank you again for your consideration.

Sincerely,
Anna Shafer-Skelton, Timothy F. Brady, and John T. Serences

Reviewer #1:

Reviewer #1: I went through the revised manuscript and the author's response, and I think the paper has improved in multiple aspects. Nevertheless, I still have a few concerns that I believe need to be addressed:

- The Introduction is now much better in terms of balance regarding the prior literature, no longer creating the impression the authors are attempting to refute Lescroart & Gallant [13] through the same setup (video) with motion control. It seems like the Abstract however has not been updated to reflect this change in framing. Especially the sentence "this raises the question…" is, in my opinion, too speculative for an Abstract – after all, the current manuscript does not directly address the low-level motion control for that study. I suggest rephrasing the Abstract to focus more on the contributions of the current work and less on the implications regarding validity of earlier claims (similar to the Author Summary).

> We appreciate this feedback. We have now updated the abstract to be more in line with the Author Summary. It now reads (p. 2):
>
> > "Surprisingly, we found evidence that a Gabor-wavelet baseline model—commonly thought of as a "low-level" or "2D" model—better fit voxel responses in areas OPA, PPA and MPA/RSC compared to a model with 3D-surface information. ~~This raises the question of whether previous findings of greater 3D information could have been due to a baseline condition that didn't model some potentially critical low-level features (e.g., motion).~~ We highlight that this difference in results could be due to differences in the baseline conditions used across studies. ~~Our findings also~~ These findings emphasize …"

- It seems the authors have not followed my suggestion to include an additional paragraph at the start of the results that summarizes the methods to better prepare the reader. I still think it would greatly benefit the accessibility of the paper to do so, i.e. to briefly summarize the fMRI experiment setup, explain that you had two behavioral tasks, etc., walking the reader through all panels of Fig 2 (which is currently skipped in text in the Intro/Results), and explain that the fMRI analysis consists of voxelwise encoding and variance partitioning.

Thank you for this reminder. We now include a more complete overview of the approach/methods at the start of the Results section to orient the reader (p. 12): In this study, we compared the performance …"

- Results: "Note that none of our results are normalized to a noise ceiling, so we cannot compare absolute magnitudes with noise-ceiling-normalized results." What prohibits you from computing a noise ceiling and plotting the results in that way, facilitating the detailed comparison?

We have now added extra detail to the Methods (pp. 42, 43) and Results (p. 18). See below for details. Because more detail about noise ceilings is now included in these sections, we have removed this sentence from the first paragraph of the results (p. 12).

To provide more explanation for our original decision not to rescale our results by noise ceilings: First, our stimulus set had 3 exact repeats for each combination of image, task, and dot pair, and Lescroart & Gallant [13] report that the method they used for noise-ceiling estimations is unstable with fewer than 5 repeats. This detail is added to the updated text at the end of *Methods: Ridge regression (p. 42)*: "For the follow-up analyses investigating the effects of noise-ceiling rescaling, …"

Second, especially because of other differences in our design, we wanted to be more conservative by not rescaling our prediction performances based on noise ceilings. Lescroart & Gallant divided their data into separate training and test sets, with both noise ceilings and the final model performance calculated from the test set. Thus, any noise present in their separate test set should affect both the noise ceiling estimates and the validation performance, which (at least if repeats were evenly distributed across validation runs) shouldn't cause any underestimation of the noise ceiling relative to model performance. Since our experiment wasn't designed to have an initial model-selection step, all runs were included in the main cross-validation loop. Our design also did not require each image's Nth repeat to appear before any image's N+1st repeat, which means the noise ceilings could be disproportionately affected by any across-run and across-session variability that the prediction performances are not subject to. We reasoned that if this were the case, we risked inflating our estimates if we used noise-ceiling-rescaled model performance, especially because the regularized regression used in the model-fitting procedure allows added flexibility compared to the noise ceiling estimation. We also reasoned that this difference in rescaling was very unlikely to cause a change in statistics or patterns of results and would primarily affect the interpretability of the rescaled *magnitudes*.

We nevertheless appreciate that readers may be reassured by seeing a calculation of the noise ceiling, even if our smaller number of exact repeats prevents us from using the same method as Lescroart & Gallant. We have used the method reported by Allen et al. [35], which we believe is less affected by differences in numbers of repeats. As expected, this version of the results was quite similar to the original version aside from magnitude. For those interested in comparing our noise-ceiling-rescaled data to Lescroart & Gallant's, we have now added a comparison of our noise-ceiling-rescaled data to our raw data (Figure 7, A-F) and we have added new paragraphs describing this analysis in the Results and Methods sections. *Results (p. 18), paragraph starting with:*

> "We also tested whether the pattern of higher Gabor-baseline vs. 3D information could have been partly due to not rescaling model performances by voxel noise ceilings, …"

Methods: Ridge regression (p. 42), paragraph starting with:

> "For the follow-up analysis investigating the effects of noise-ceiling rescaling …"

We want to note that after rescaling by noise ceilings, while one ROI did show significant unique 3D information for one of the 3D models (see Figure 7D), the *magnitude* was visually indistinguishable from the original analysis, and the pattern of greater unique Gabor information was even more reliable than before. To add some intuition for these small differences, if the voxels with the lowest noise ceilings within an ROI systematically had higher performance for the 3D vs. Gabor-baseline models, rescaling could have re-weighted the contributions of those voxels to be higher. In theory, if this relationship were strong enough, it could have even reversed our pattern of stronger Gabor-baseline performance. However, we find little or no change in relative model performance at all. This converges with our flat-map visualizations showing that it was very rare for 3D performance to be greater than Gabor-baseline performance, even for smaller regions within ROIs. This gives us even more confidence that neither our results nor [13]'s results are likely to be an artifact of decisions about noise-ceiling rescaling.

In updating our figures, we also realized that since we didn't rescale by noise ceilings in our main figures, it was difficult to visually judge the individual-subject data points in some plots, especially in scene regions. In Figures 3, 9, and S2, we have now plotted scene regions on a separate set of axes for legibility. In addition to clearly labeling each set of axis ticks, we have explicitly noted this axis difference in the figure caption.

We also note that, especially since Lescroart & Gallant's flat maps were FDR-corrected, using a noise-ceiling cutoff for flat-map voxel selection allows for a closer comparison to their data. We have now used a noise-ceiling cutoff to select voxels to display in the flat maps, after which we used an FDR-correction. (See "minor points" below for a more complete discussion of unique 3D variance anterior to RSC.)

- For the surface flatmaps: Analogous to the ROI analyses, in addition to the unique partitions, please also show full/raw correlations for the 2D and 3D models for each participant, so those can be compared to prior literature.

We have now included full/raw correlations for all 2D and 3D models for each participant. For participant S01, these are now included in the main-text Figure 5, as they are for the variance-partitioning surface flat maps (now Figure 6) and as in ref. [13]. The respective S02-S08 raw-correlation plots are now included in Figure S3, as they had been for the analogous variance-partitioning plots (now S4), and as they are in ref. [13].

One citation that the authors missed but that seems particularly relevant in this context is Dwivedi et al., PCB 2021, who report differential mapping of DNNs trained on the Taskonomy dataset on 2D vs 3D tasks in dorsal vs. ventral ROIs.

Thank you for pointing this out. We appreciate the opportunity to contextualize our results in terms of the Dwivedi et al. (2021 PCB) results, which compared prediction performances for features of DNNs that were trained on 3D vs. 2D tasks. This fit nicely into the edits we made to clarify the Discussion (see also the point below describing these changes). This portion (p. 32) now reads:

> "Two recent studies have made headway in this direction. Dwivedi et al. [29] and Wang et al. [30] tested how well human brain responses could be predicted by the internal representations of several DNNs [15] that were trained to predict 3D properties of scene images (e.g., surface normals) vs. 2D or semantic properties of images (e.g., 2D edges or scene category, respectively). They each found promising relationships with brain representations for features from 3D vs. 2D DNNs, setting the stage for future work to more directly interpret the content of these feature sets."

- Upon reading the paper again, I realized the study design seem geared towards testing a different hypothesis; perhaps regarding the spatial locality of task-relevant 3D information? It would be good to explicitly mention this as now there is no motivation provided for several aspects of the experimental design.

We now make this more clear in the relevant sections of the Results and Methods, and we add an additional section at the end of the Methods: Deviations from original analysis plan.

Updated text in the first paragraph of the Results (p. 12):

"On each trial, participants were asked to make one of two judgments: …"

Updated text in *Methods: Stimuli and Tasks* (p. 37):

"This experiment was originally designed to test the robustness of greater 3D-vs 2D-model performance to changes in task and spatial attention."

New section: *Methods: Deviations from original analysis plan* (p. 43):

"This study was designed to test whether the advantage for the 3D model vs. the Gabor-baseline model generalized from ref. [13] to a new stimulus set and, if so, …"

- Discussion; I appreciate the newly added sections on 23-26, aiming to clarify the difficulty of disentangling 'low-level' vs. 'high-level' features in scenes. However, this section makes the Discussion section quite lengthy, and in the end I'm not quite sure what the authors want to say; are they arguing against using variance partitioning as a method (cf, page 25/26 "Without a more thorough understanding…")? The current Discussion both praises and criticizes the use of such methods on naturalistic stimuli (in contrast to controlled stimuli) but doesn't clearly outline what should then be improved (note that the reference to task manipulations in the last paragraph seems quite out of place, given that no task effect was found on the encoding model performances here). How do they envision we obtain such more thorough model understanding – do we need a different paradigm for this? I suggest shortening and consolidating these sections around key points the authors want to make.

We have now made these sections more concise to keep the Discussion more focused on the key points we want to make. This includes outlining the potential paths forward that we see and more directly clarifying the roles for hand-designed vs. naturalistic visual stimuli, within them. For example:

p. 32: "**How can we meaningfully characterize the format of higher-level visual information?** Model comparison remains a critical tool for precisely quantifying the degree to which different types of information are present across the brain. The present work demonstrates that the full benefits of this approach depend on better understanding the information captured by each model and how model features covary…"

p. 32: "One potential path forward is to focus on finding the most theoretically meaningful cutoff between "higher-level" 3D spatial features and the corresponding "lower-level" 2D features used as a baseline condition. …"

p. 33: "A second potential approach, either before or instead of finding a binary cutoff between "lower-level" and "higher-level" feature sets, would be to develop a better

understanding of how features covary in naturalistic visual input. To the extent that certain feature sets reliably covary, even features that seem to be “lower-level” could be used by the visual system as 3D or other “higher-level” information.”

Minor comments:

- Small note on Introduction: It wasn’t immediately clear to me what ‘To this end’ at the start of the second paragraph refers to: to ‘disambiguating representations’, or ‘studying the content of scene information’? It would be good to explicitly spell out the goal here.

This comment highlighted that “to this end” was redundant with the later portion of the sentence, so we have shortened it to (p. 4):

“Here, we focus here on assessing whether these areas contain information about surfaces and 3D spatial layout per se rather than other visual properties of scenes.”

- Results Page 12 “We found similar magnitudes of cross-validated prediction performance” – the way this is phrased made me initially (I hope erroneously) think that the main results reported in Fig 3 are not cross-validated. I think the authors are trying to emphasize the ‘magnitudes’ part, but since the sentence before it does not mention ‘cross-validated’, it seems like that part is new. Also, it would be good to explicitly say ‘similar magnitudes … as in reference [13]’.

Thanks for catching the confusing phrasing! All results shown have indeed been cross-validated, and we have clarified this sentence to read (p. 14):

“The magnitude of the raw cross-validated predictions was similar to Lescroart & Gallant’s [13].”

- Results: In the pdf I received to review, the text-embedded Figures 4 and S2 were of poor quality, with blurred text and completely illegible axes labels for panels 4B-C. Also, the separately uploaded .tiff files looked blurry in the pdf (but those other figures then again looked OK in-text). Please ensure sufficient figure quality by exporting figures with high resolution, and evaluating converted pdf quality.

Thank you for catching this. We have now corrected the steps of our converting and embedding process so the figures do not become blurry when embedding them. We have additionally re-plotted figures so that the text sizes on some axes are much larger and so that the aspect ratios enable larger text sizes.

- In the revised Results section, the panels of Figure 4 are discussed in reverse order. Please either rearrange the figure or change the text to ensure the text introduces each panel in the correct alphabetical order.

We have now split Figure 4 into multiple figures, each including the corresponding model-performance results and both versions of the variance-partitioning results. This corresponds to the new Figures 4, 8, and 9. This has enabled us to include figures in the same order as they appear in the text.

- Figure 5 is currently not discussed or described in text.

We have now added a discussion of Figure 5 in the Results section (p. 20):

"However, individual-participant surface maps (Figures 5, 6, S3, and S4) show that very few voxels in visual cortex had higher 3D vs. Gabor-baseline model performance, …"

- The quality of the flatmap figures can be improved. Because of the use of black backgrounds and showing the Gabor model performance in dark blue, vertices with significant performance are hard to discern – it might be much clearer when using a bright color. The ROI boundaries have varying colors/color intensities that are not explained in a legend (initially I was mistaking the purple ROI boundaries in Fig 5 for R2 by the Gabor model, as this is how it is indicated in the bar plots), and the text in Figure 5 is again somewhat blurry (curiously, Figure S3 looks much better). The error bar labels for the flatmaps show overlapping text labels and are of low resolution – it looks like it was made via a screenshot from a GUI. It may help to edit or remake such figure elements in a separate figure editing program (e.g. Illustrator or Inkscape) to ensure good legibility.

Thank you for this feedback. We have now: 1) Changed the backgrounds to be white instead of black, 2) lightened the gray brains so the blue overlays are much more visible, 3) changed the ROI boundaries to all be white so they don't cause confusion or detract from the overlays, and also added a sentence in the caption explaining that the white outlines are the ROI boundaries, and 4) re-exported and edited the color-scale for each of the overlays so that they are more legible. We have also now saved each of these figures as a PDF before embedding in-line into the marked-up manuscript, and we have checked that they are legible and not blurry in the final exported marked-up PDF.

- Figure 5 and S3: All participants seem to show some unique 3D variance anterior of RSC. Do the authors want to comment on this? Is this noise, or potentially meaningful?

We had previously plotted raw correlation values for visualization purposes only, and we have now thresholded prediction performances and variance-partitioning results based on FDR-correction. This was made possible by voxel selection that was based on noise ceilings for each voxel, which reduced the number of voxels to be more tractable. In the previous version, the warm-colored voxels anterior to RSC appeared to be part of a larger pattern of speckled warm-and-cool noise that wasn't always visible from the angle of the screen shots. After thresholding based on an FDR-corrected $q < 0.05$, this warm-and-cool speckled noise is no longer present, either in the screenshots or in the rest of the surfaces (e.g., Figure 6).

- Discussion page 24 on baselines "This is especially true when…" but [13] still found a unique contribution of 3D despite picking the 'best baseline' in this way, so I don't see how this supports the point regarding the flexibility of baseline models here? Please clarify.

Thanks for asking about this. We believe this is because of the separate point that the baseline condition in their experiment did not attempt to model all relevant low-level information in the stimuli, leaving out motion. (And we have heard anecdotally that a motion-energy model performs much better than other models in that data set.) However, this comment has made us realize that the connection to [13] is tangential to the point of this paragraph, so we have removed it since it would require additional explanation to be clear.

Reviewer #2: *This revised manuscript includes a number of improvements over the original submission, primarily in how clearly the data are presented, with an improved motivation for the study, and with further elaboration on some points in the discussion.*

However, I don't feel that the authors have really addressed the only major comment I raised in my original review, namely that they fail to find evidence of any differences between ROIs. The main result in this study is essentially a null effect (no difference across ROIs in the relative performance of 2D vs 3D models), which argues against conclusions of previous studies, and the methodological approach is relatively complex, making it harder to intuitively understand its limitations. Given these factors, it would be reassuring if the authors could demonstrate that their data and modelling approach can be used replicate other known differences between regions, such as their dependence on spatial frequency. Being able to show that they can reveal differences across ROIs for coding of an alternate stimulus dimension would show be a helpful way to demonstrate that the 'null result' of no difference across ROIs is less like to be attributed to the data have insufficient signal to noise in higher level areas, or the modelling approach not being sensitive to differences between ROIs for some reason.

Thanks for the extra clarification on this point—it seems that we may not have understood your original concern fully. We now understand your concern to be "the main result in this study is essentially a null effect (no difference across ROIs in the relative performance of 2D vs 3D models), which argues against the conclusions of previous studies". First, we would like to clarify that the main results in this study are the *significantly higher* Gabor vs. 3D comparisons *within* scene-selective ROIs. While we have now run an across-ROI ANOVA for our main model comparison and found a highly significant ROI x Model interaction, these results do not support or refute our main conclusions (nor did [13] report any across-ROI stats). We had previously understood the original point as being in agreement with this statement because of the part saying that any lack of differences across ROIs "doesn't undermine the main conclusions, since they are based on the relative performance of the 3d vs. 2d models within each ROI". Because of this, we had not previously tailored our response to this concern.

We now believe we understand this concern more fully, and we have addressed this point by A) adding changes in the text and figures to clarify that our conclusions depend only on within-ROI comparisons; B) adding a supplementary ANOVA showing significant across-ROI differences for our main analysis (We apologize that without running this analysis, it had initially appeared that we had found null across-ROI effects!); C) making further improvements to the Discussion section to clarify our fitting procedure and the details of the Gabor-wavelet model and to explicitly contextualize our results within a broader range of previous literatures, including showing they are consistent with the known properties of the visual system.

A) Text changes to clarify that we are not drawing any conclusions from null effects, nor from effects across ROIs:

First, we have edited the subsection header and text in the Discussion to emphasize the significant within-region model comparisons and to make it more clear that our conclusions do not depend on any null effects (p. 25):

> "**More information uniquely attributable to a Gabor-wavelet baseline model than to 3D scene-surface models:** In each scene region, our main variance partitioning analysis revealed significantly greater information uniquely attributable to the Gabor-wavelet baseline model than to either scene-surface model. This was the case in almost all of our follow-up analyses as well, with the exception of the task-breakdown analyses, where for several comparisons within RSC, Gabor model performance was numerically but not

significantly higher than 3D model performance. We also found little to no evidence that 3D scene surface features uniquely explained any activity in scene-selective cortex, despite consistent above-chance performance of the Gabor models."

Because readers may want to quickly skim figures for the highlights, we have also added within-ROI statistics to all bar plots so that this point is reinforced even with a quick glance at the figures. To further improve the legibility and emphasis on these within-ROI statistics, we have also adapted our plotting functions to increase the size of the significance markers. In order for readers to better ascertain the data quality in the scene regions, we have plotted them on a separate axis with clear y-axis ticks so that both the relative magnitudes and the individual-subject data points are more legible.

Next, we emphasize that it is our *within-ROI* comparisons that argue against conclusions of Lescroart & Gallant [13]. Indeed, Lescroart & Gallant [13] also do not report any across-ROI statistics and do not draw conclusions about statistical differences across ROIs. We have also further clarified that we have found *significant* within-ROI effects in the *opposite direction* of their Gabor vs. 3D comparisons in each ROI, and that we are not reporting null results across ROIs (text reproduced below):

> *Results (p. 14):* "For scene-responsive regions OPA/PPA/RSC, we found that while prediction performance was significantly above chance for all models (all ps < 0.014; Figure 3A and Table S2A), the Gabor-wavelet model unexpectedly outperformed the 3D models (Figure 3A; all ps < 0.026) in each scene region, demonstrating significant effects in the opposite direction as in ref. [13]. (Note that while neither our nor ref. [13]'s conclusions depend on across-ROI comparisons, we include a supplementary across-ROI ANOVA (Table S1) to reassure readers that power in our analysis approach is adequate to detect such differences."
>
> *Discussion (p. 26):* "With this change, we observed that the Gabor-wavelet baseline model generally outperformed the 3D model within each scene region, demonstrating significant results in the opposite direction as in ref. [13]."
>
> *Discussion (p. 26):* "… the higher performance of the 3D-surface model within each scene region in [13] could …"

See also sub-response C), below, for clarification of how our results are consistent with the previously cited spatial-frequency results.

B) Supplemental ANOVA showing extremely reliable across-ROI differences in model performance:

Even with the clarifications provided above, we welcome the opportunity to reassure readers that our fitting approach does not lack the power to detect any across-ROI differences. While it may have appeared that we found a null across-ROI result in the previous version of the manuscript, we had in fact not tested across-ROI differences because they were not directly related to our main point. However, we now understand that from the way the paper was written, readers could have easily taken this lack of *mention* of across-ROI statistics as a null result. We have now added a supplementary analysis (Table S3): a repeated-measures ANOVA on the model performances across ROIs. Not only do we find extremely reliable main effects of ROI and model, but critically we also find an extremely significant interaction between the two. While the Gabor model is the strongest model in every region, this interaction reflects the magnitude of the Gabor *advantage* decreasing from early visual regions to scene regions (see the corresponding plot that is reproduced below the stats table).

ANOVA Results:

	F Value	Num. DF	Den. DF	P-value
ROI	99.644375	5.0	30.0	9.363692e-18
Condition (Model)	749.430270	2.0	12.0	2.510390e-13
ROI x Condition intxn.	99.208901	10.0	60.0	1.780918e-33

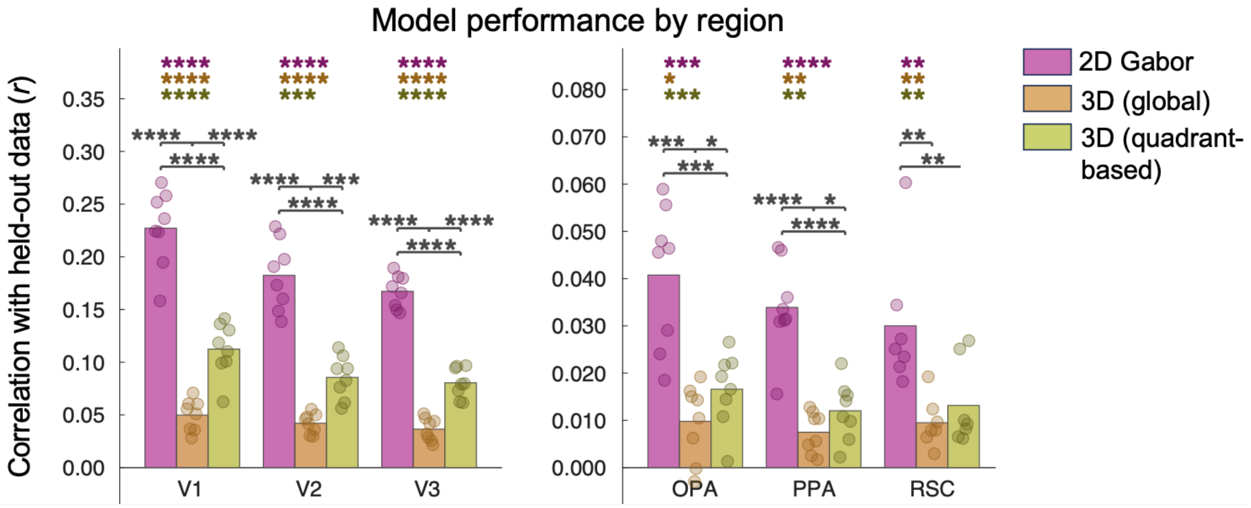

C) Further clarifying details of the Gabor-wavelet model and contextualizing our results within testing different types of spatial-frequency features inside and outside of scene regions:

Even with the reassurance that our approach has adequate SNR to detect across-ROI differences, we agree that the methodological approach is relatively complex, and we have taken this opportunity to further clarify it in the Discussion sections so that this work will be more relevant and accessible to a wider range of readers. One thing that makes this modeling approach complex is the structure of the Gabor-wavelet model. Rather than independently varying the spatial frequencies at each location tested in the visual field, a Gabor-wavelet model captures a dependence between these features: higher spatial frequencies are tested with smaller spatial fields. This model has performed quite well in visual regions, likely because this dependence captures the known properties of the visual system in a relatively compact set of features. In contrast, for work in which the goal is to quantify information about *specific* spatial frequencies, a Gabor-wavelet model is not the best approach. We have now added more explanation of Gabor-wavelet model features to the section *Methods: Feature sets: Gabor wavelet baseline model* (p. 40):

> "Note that there is a dependence between the spatial frequency and tile size: lower spatial frequencies are used for smaller tile sizes."

We are also enthusiastic about the opportunity to further contextualize our results within the broader literature, including studies that test selectivity for spatial frequencies and other features across visual cortex.

To address both of these objectives, we have added a new paragraph in the Discussion (p. 29):

Our results are also consistent with results reporting variations in the sizes of voxel receptive fields (vRFs) and spatial-frequency selectivity within and across regions. First, scene regions tend to have larger voxel receptive fields (vRF's) [e.g., 20] than regions of early visual cortex [e.g., 21], and our Gabor-baseline model includes smaller receptive fields than both the Global and the Quadrant-based 3D models. While these vRF properties do not make predictions about 2D vs. 3D feature differences per se, they do predict a *smaller advantage* for the Gabor vs. 3D models in scene regions vs. EVC regions when considering the regions of visual space captured by the features of each model. In a follow-up ANOVA, we found a significant ROI x Model interaction supporting this across-ROI prediction (Figure 3; Table S2). Several previous studies have also found selectivity profiles for spatial frequencies in scene regions that are consistent with these regions being well described by a Gabor model [22-24]. Other work has found that while *radial frequency* (capturing curvature information) appears stronger in early- to mid-level regions vs. higher-level regions, *spatial-frequency* information similar to that captured by our Gabor-baseline model is relatively preserved across all regions [25]. Our observation that scene-selective regions are well described by Gabor model is thus compatible with this finding and adds to the substantial literature on spatial-frequency selectivity in scene-selective cortex.

While Reviewer 2 did not explicitly indicate a perceived conflict between our work and either Goddard & Mullen (2020) or Salmela et al., (2016; ref. [25] in main text), we pre-emptively add more detail about why our results are compatible with each, since both papers were cited in a previous round of review. (We figured it was better to add an explicit explanation, just in case it helped with any additional concern we might have missed! Apologies if any or all of this explanation is superfluous.) Because Salmela et al. (2016) includes a similar spatial-frequency condition to our work, we have added this citation to the main text for any readers wondering how our results compare.

Goddard & Mullen (2020) presented participants with radial sine-wave grating stimuli (appearing as a series of concentric rings) and found a significant decrease of this form of spatial-frequency information (rings nested closer together vs. farther apart) in three higher-level ventral regions (V4, VO1, and VO2) vs. EVC. First, we note that, even to the extent that these stimuli captured a similar type of spatial-frequency information as our Gabor-wavelet features, spatial frequency was not manipulated independently of temporal frequency, preventing any comparison to stimuli that did not include the same conditions. Second, due to SNR differences both across regions and across scanner protocols, any across-region differences attributable to spatial-frequency per se would only be meaningful in reference to the other temporal and chromatic conditions included in that data set, as is standard for the interpretation of fMRI data. None of the above studies examining spatial frequency in scene regions, including ours, included temporal or chromatic features, and none of the above conflict with Goddard & Mullen (2020). Goddard and Mullen (2020) also did not localize PHC-1 or PHC-2, the retinotopic regions that primarily overlap with PPA (Arcaro, McMains, Singer, & Kastner, 2009). V4, VO1, and VO2 also do not overlap with any other scene regions (OPA, MPA/RSC). Because different higher-level regions can have different feature selectivities, a hypothetical of decreased spatial frequency information in V4, VO1, and/or VO2 would motivate future work expanding similar analyses to scene-selective cortical regions. This is an interesting topic for future work.

Salmela et al. (2016) presented participants with stimuli that parametrically varied both *radial frequency* (capturing a type of curvature information) and *amplitude*. They reported that *radial frequency* information (RFC) was reported in V2d, V3d, V3AB, and IPS0, but not hV4 or LO. This radial frequency information, capturing curvature, is different than the spatial-frequency information captured by single, un-curved, oriented sine-wave gratings in Gabor-wavelet models. Interestingly, Salmela et al. (2016) also calculated spatial-frequency

spectrum (SF-spectrum) information, which is the condition most similar to our Gabor-wavelet baseline condition. They found that "…SF-spectrum and contrast energy related representations were found across visual areas but without similar specificity for visual areas that was found for RFC." These results are consistent with our findings that the Gabor-baseline model is relatively preserved across all regions, although note that the relative performance vs. other conditions may be different according to the other conditions tested.

Minor points

- some figures remain too low resolution to read, such as Figure 4

Thanks so much for this feedback. We have now corrected two steps of our figure exporting and embedding process. We have also updated our plotting scripts to automatically output larger text sizes for all figures, and we have confirmed their legibility after embedding them into the marked-up PDF.

- the new Figure 5 is not referred to or discussed in the body of the Results text.

Thanks for catching this. We have now described Figure 5 (currently Figure 6) in the body of the Results text.
