# Supplementary material for: A 2D Gabor-wavelet baseline model out-performs a 3D surface model in scene-responsive cortex
Source: PLoS Comput Biol. 2026 Feb 2;22(2):e1013888. doi: 10.1371/journal.pcbi.1013888 (PMC12880747; doi:10.1371/journal.pcbi.1013888)
Supplement: Attachment — Submitted filename: response_to_reviewers_clean.docx [file pcbi.1013888.s009.docx]

Dear Dr. Kietzmann and Dr. Martin,

Thank you for your feedback on our manuscript "A 2D Gabor-wavelet baseline model out-performs a 3D surface model in scene-responsive cortex" (PCOMPBIOL-D-24-00406). We greatly appreciate the effort of yourself and reviewers, and we believe the paper is significantly improved as a result of this feedback. In our point-by-point response below, we highlight the changes we made in our manuscript to address the concerns of the reviewers. In our revised manuscript, we have highlighted the changed sections in **orange** for clarity.

We hope that the present revision may be viewed as a substantial improvement that will be acceptable for publication. Please don’t hesitate to contact us if you have any further questions, and we look forward to hearing from you. Thank you again for your consideration.

Sincerely,

Anna Shafer-Skelton, Timothy F. Brady, and John T. Serences

***Reviewer #1:***

*This study presents an fMRI experiment which utilizes voxel-wise encoding models to disentangle the neural representations of 2D vs. 3D information in BOLD responses to scene images in scene-selective cortex in the human brain. The 2D model is a Gabor wavelet model and the 3D models are derived from ground-truth distance and surface maps in the Taskonomy dataset. Stimuli were chosen from the larger Taskonomy dataset by minimizing representational distances between the different model features. The authors explicitly indicate that the central message of the paper is that their results contradict prior work that claims more (unique) representation of 3D scene information in scene-selective cortex than 2D information, focusing strongly on one high-profile paper by Lescroart & Gallant (2019) who used movie stimuli and similar models (but also several other studies, as highlighted in Figure 1). They argue that the previous claims of 3D representation may be due to co-variation of 3D cues with motion, or the use of weaker 3D baseline than those employed here.

MAIN COMMENTS

The study is well designed – the use of the Taskonomy images is clever given the availability of 3D ground truth data in those static images, and the fMRI experiment is highly powered, with carefully considered tasks. The model fitting procedure and ensuing analyses appear solid, and many of the points made by the authors are fair and will I think be important to communicate the field of scene perception research.*

Thank you for your positive assessment of the work and for your constructive feedback.

*Stylistically, I am a bit unsure though about whether the strong contrast that is drawn with the Lescroart study is completely fair given that the authors here opted to remove motion information entirely (by using static stimuli) rather than running a new study with movies with the same or similar stimuli and including a motion-baseline model – if in that case no unique variance for 3D information is found, it would be a very strong non-replication. I’m not suggesting the authors run this study, but it might be worth setting up the Introduction a bit differently, so that the reader isn’t ‘disappointed’ when they find that the current study in fact didn’t use video (as I was when I arrived at page 7 “In the present study, rather than attempt to expand a baseline model for the complexities of video stimuli, we instead removed…”). The Introduction seems set up to present that.*

We greatly appreciate this feedback--we agree that we don’t want the manuscript to read as (or set up the reader to expect) a strong non-replication of Lescroart & Gallant (2019). We have incorporated edits throughout the text that we hope can shift the emphasis a bit more towards general theoretical points about the challenges and ways forward for studying “higher-level” info that necessarily covaries with “lower-level” features.

First, we have framed the Lescroart & Gallant work more in the context of the overall theoretical challenges in the field. Second, we have shifted emphasis from Lescroart & Gallant’s choice of baseline model in particular, onto the more general relationship of the baseline model to the cues present in the stimuli. We also took opportunities to decrease suspense around some aspects of our findings, which we felt had too much of the tone of a (non-)replication. Selected text from the relevant paragraphs (pp. 6-8) now reads (underline indicates new text):

“This in-principle issue might be side-stepped, however, if a model of the 3D structure of a scene performed decisively better than a set of high-performing baseline models. Lescroart & Gallant [13] constructed … The high performance of the 3D vs. baseline models appeared to resolve the theoretical issues described above. … However, the combination of moving stimuli with a static Gabor baseline model raises concerns: … Note that this possibility would not by itself argue against the presence of 3D information in these regions—instead, it would highlight the importance of building a more nuanced picture of covariation between “3D” and “2D” feature sets (for a more detailed explanation, see *Discussion: Implications for the visual system*).

“In the present study, we sought to characterize the performance of 3D scene-surface models [13] and their relationship to the orientation and spatial-frequency features they covary with in the natural world. First, we wanted to ensure that our baseline model encompassed the full set of low-level features we wished to discount. We could have done this by either A) using an expanded baseline model to account for the low-level motion features present in video stimuli or B) using static photograph stimuli instead of video stimuli so that it was unnecessary to include low-level motion information in our baseline model. We chose to use static image stimuli with a static gabor-wavelet model (capturing orientation and spatial-frequency information), in part to maintain more contact with prior work that used static orientation and spatial-frequency features as a baseline [e.g., 9, 11, 12]. In addition, the photographs allowed us to characterize the performance of 3D scene-surface models using natural images, as opposed to the computer-rendered stimuli used in prior work. While this choice sacrifices one aspect of “naturalness” of the stimulus set (motion), the photographs appear more “natural” in another sense, in that they are more lifelike than stills from a computer-rendered video.”

This comment also highlights that it was our intention to convey two points with this manuscript: 1) the point about the static gabor model being an unfair baseline that works *in favor* of Lescroart & Gallant’s (2019) conclusions (explained in the text above), as well as 2) a point that Lescroart & Gallant’s strategy of choosing the highest-performing baseline model works *against* their conclusions—this is specifically because it is likely that “lower-level” features (e.g., the patterns of orientation and spatial frequency quantified by a GIST or Gabor-wavelet model) necessarily act as cues to the corresponding “higher-level” features (e.g., 3D surface configurations). The second point is a fundamental challenge in the field, which we now devote more attention to in the discussion (“Implications for the visual system”, p. 22).

*The Results section is also oddly short. It is clear the authors want to make a punchy point and put all the ‘details’ in supplement, but I don’t see why that’s necessary for this journal as there are no page restrictions to report the analyses in the full text. It also doesn’t allow the reader to see a bit more of this potentially very interesting dataset without making extra effort to download the supplement. (NB The Supplementary figures appear to be screenshots of Matlab figure windows, some of which are not well readable (e.g. S3). I do not think this should be considered acceptable for publication, especially not since important additional analyses are presented in the Supplement).*

We have now moved the supplemental from the supplement to the main text (Figure 4 A-C, p. 16), and we have made the figures publication quality.

*Also, the sole Results figure (Fig 3) is just bar plots: for a more comprehensive comparison with Lescroart & Gallant (2019), it would be good to include a visualization of encoding model performance on flatmaps along with ROI definitions (see also next point).*

We have now created individual-subject surface maps, for better visual comparison with Lescroart & Gallant (2019). Because we share Lescroart & Gallant’s concerns about aligning across subjects, specifically for OPA (Lescroart, VSS 2024), we visualize results only in native surface space. As in Lescroart & Gallant (2019), our first participant’s surface maps are included in the main text, and the remaining participants’ maps are included in the supplement (pp. 39-41).

*Although the authors clearly already thought deeply about their distinctive results from Lescroart & Gallant (2019), and did several additional analyses in an attempt to rule out alternative explanations, I think there may be one additional factor that could potential play a role, namely the ROI definitions: in Lescroart et al. any voxels in PPA, OPA and RSC that overlapped with retinotopic maps were excluded (I think this is the general custom in the Gallant lab). Although here the authors test for selecting subset of voxels, I don’t think the analyses included a systematic removal of only those voxels that overlapped with the same retinotopic maps as in L&G. Here, the authors also use mutually exclusive ROIs, but mapped out only V1-V3, while L&G included higher-order maps such as V4, V3AB, LO and V7. It’s perhaps not very likely that any of these will overlap with PPA (which does overlap with retinotopic maps anterior to V4, see Arcaro et al., 2009) but definitely some will overlap with OPA (see Silson et al., 2016). In other words, it seems pertinent to run a control analysis where OPA/PPA are defined without any retinotopic overlap similarly to Lescroart and colleagues to exclude an explanation of the difference in results between the studies based on the inclusion of retinotopic voxels.*

Thanks for this suggestion. We have re-run a version of analyses with hV4, V3AB, LO1, LO2, and V7/IPS0 additionally excluded from our scene regions. Since this change yields virtually identical plots, we have added a sentence to p. 6 describing this analysis and have included the plot as Figure S2 (p. 38). We also think this point raises interesting speculation and future directions for the relationships and response properties of these overlapping regions (especially for OPA, which has the most potential overlap). We have added this to the Discussion (p. 23).

*MINOR COMMENTS

Order of presentation: Some of the Results sections could not be followed well without reading the Methods first; it would help readability to present Methods first, or to provide more context on the Results, e.g. to preface some more of the methods in Intro or start of the results. For example, from just Fig 2 we can’t tell if the method is RSA or voxelwise encoding or something else.*

We have now included additional methods information in Figure 2’s caption and in the results, including additional details in analysis methods. We also include more details in the body of the Results (highlighted in orange, pp. 11-14).

*Same for the selection of stimuli, it is not obvious from the caption of Fig 2 between which models the RDM correlation was explicitly minimized (from the Methods, it becomes clear it was never 2D vs 3D but that just turned out to be so as a side-effect of minimizing 3D global vs. 3D quadrant? Isn't that interesting in itself actually?).*

We have now added additional details to the Figure 2 caption (pp. 10-11) to better clarify the stimulus selection procedure:

“(C) The 300 stimulus images were chosen to minimize 3D-global- vs. 3D-quadrant-based-model RDMs. First, an initial set of stimulus images was chosen iteratively (similarly to Groen et al., [16]), from a pool of images with maximally different depth information in the image’s 4 quadrants. The resulting initial image set had an RDM correlation of 0.7 between the quadrant-based and global 3D models. Next, we replaced each of the 300 stimulus images with the image from 1000 random choices that resulted in the lowest RDM correlation. This stimulus selection procedure incidentally yielded a low correlation between the Gabor-wavelet baseline model and each of the two 3D models. While our main analyses used voxelwise encoding models, we used RDMs for our stimulus selection procedure to quantify feature variation across images.”

*The Results section on voxel selection (page 12) it is unclear what ‘our pre-determined voxel counts’ refers to (without reading Methods).*

We have now updated this text (pp. 12-13) to be more descriptive. It now reads:

“First, it could have been the case that by including up to 200 voxels in each category-selective ROI (possibly a less stringent cutoff than other studies, e.g., [13]), we might have ended up including voxels with more selectivity to lower-level vs. category-selective features…”

*Discussion P18: ‘..local affordance (arguably 3D); Bonner & Epstein 2017 never explicitly argued that their path drawings implicate a 3D representation, I think? So it would be good to substantiate this argument a bit more.*

This comment matches our reading of Bonner & Epstein (2017). We have incorporated edits throughout the manuscript to clarify the relationship of Bonner & Epstein’s (2017) findings to the other studies that more explicitly argue for a 3D representation. The most substantial of these edits is on page 21:

“First, Bonner & Epstein [6] investigated local scene affordances, or representations of the parts of a visual scene that a viewer could traverse within the depicted 3D space. They found higher performance for GIST features than for local-affordance (arguably 3D) information. Note that while this work did not explicitly test the presence of 3D surface information, local-affordance information is related to 3D surface information since configurations of surfaces within a scene constrain where humans are able to walk.”

*Methods Page 26: It would be good to say a bit more about how the ground-truth for the Taskonomy image maps were obtained to construct their training data. After all it is very important for the conclusions of this study that the 3D model is in fact accurate in representing the 3D structure of these (real-world) images.*

We have now added more information to the methods section (p. 31): “These distance and surface-normal image maps were generated from images taken with RGB-depth sensors. Information from multiple camera rotations and viewpoints of the same indoor space was used to improve the accuracy of these individual measurements.”

*Methods Page 28: The acronym ‘VOI’ is introduced here without explanation, is this the same as ROI or something different?*

We have now changed all “VOI”s to say “ROI” for consistency. (Because our analysis is in native volume space, we had sometimes slipped into calling them “VOI”s, but we agree calling them ROIs is more clear.)

*Figure S2: what about dot task only performance? Was there perhaps an overall reduction in correlations then? No difference in terms of model explained variances?*

Performances for both tasks were strikingly similar, even for individual-subject data points. We now plot model prediction performance for both tasks side by side in Figure 4A (p. 16.)

*LANGUAGE/TYPOS

P8 ‘While we also manipulated… there seems to be an ‘of’ lacking here in this sentence (the spatial extent of?)*

This detail has now been moved from the intro to the methods and rephrased: “While not analyzed here, each image could appear with one of three different pairs of dot locations with varying spatial extent.” (p. 29).

*There are in-line bold headers/summary statements in the beginning of the Results (page 11) and Discussion, that do not always fit neatly in the flow of the text.*

First, we have removed the headers from the Results section. Second, we have re-worked the Discussion section (pp. 17-25), and we think the headers are now less disruptive to the flow.

*Author names in citation 20 (Groen et al., 2016) is not correctly formatted.*

We have now corrected the author names in citation 20 to read: Groen II, Ghebreab S, Lamme VAF, Scholte HS.

*References:

Silson, E.H., Groen, I.I.A., Kravitz, D.J., and Baker, C.I. (2016). Evaluating the correspondence between face-, scene-, and object-selectivity and retinotopic organization within lateral occipitotemporal cortex. J. Vis. 16, 14.

Arcaro, M.J., McMains, S.A., Singer, B.D. and Kastner, S. (2009). Retinotopic organization of human ventral visual cortex. J Neurosci 29(34), 10638-10652.*

***Reviewer #2:***

*This paper by Shafer-Skelton and colleagues, presents a body of fMRI data which nicely complement the existing literature on how 3D scenes are represented by the responses of ventral visual cortex. Their main conclusion is that previous reports of responses capturing 3D scene structure may not have adequately ruled out an alternative based on lower-level visual properties, providing a timely contribution to this research question. A nice feature of this paper is that the authors checked that their main findings were robust across different methodological decisions, and the specifics of the models used.

The paper is well written, the data are carefully presented, and the main claims are supported by the data.*

We appreciate this positive feedback.

*My only substantial point is that the results would be more convincing if there were a model that better accounted for responses in the higher level areas (OPA, PPA, RSC) than the early visual areas (V1-3), and/or if the data demonstrated any qualitative differences between ROIs. In the current data, as presented in Figure 3, the overall impression is that signals are much weaker in the higher level areas, and that there aren’t any substantial differences in feature response that have been identified with this dataset. This doesn’t undermine the main conclusions, since they are based on the relative performance of the 3D vs 2D models within each ROI. The authors note that ‘magnitudes across ROIs are not interpretable, but patterns within ROIs are’ – I understand the claim here, but the magnitudes might reflect SNR. Even if there are no models that perform better in accounting for responses in higher-level ROIs than early ones, it would be helpful to demonstrate that there is enough signal to detect selectivity differences between the ROIs. One prediction that could be useful here is that higher level ventral areas tend to show less dependence on spatial frequency (e.g. https://doi.org/10.1371/journal.pcbi.1004719 https://doi.org/10.1016/j.neuroimage.2020.116780). If the current fMRI data showed a similar trend it would at least demonstrate that the dataset have sufficient SNR to detect differences between ROIs.*

We agree that the magnitude differences across ROIs are likely due to SNR differences, as is typical between early visual cortical regions to category-selective regions like OPA, PPA, and RSC. As the reviewer acknowledges, these likely SNR differences don't affect the conclusions we draw from the data we found. We also want to underscore that the comparisons of models within regions are quite reliable, except where noted in the text. This has also reminded us that since we plot our raw model performance rather than performance *normalized* by each region’s noise ceiling, our pattern of magnitudes across regions may appear more atypical, even though it is not.

We have added text clarifying this in Figure 3, as well as in the Methods section (p. 32): “We did not normalize by a noise ceiling for any of the values reported here. This means that magnitudes of effects *across* ROIs may vary due to factors like SNR differences across ROIs. Because of this, we focus on the reliability of effects *within* regions.”

*Minor queries:
-Top of page 8: I think I’m missing something here, but it sounds as though they’re saying that the baseline model they’ll include is similar to the Gabor model, but now encompasses SF and orientation – in what way would a Gabor model not include SF and orientation?*

We have updated the structure and wording in this paragraph to be more clear. The expanded section now reads:

“First, we wanted to ensure that our baseline model encompassed the full set of low-level features we wished to discount. We could have done this by either A) using an expanded baseline model to account for the low-level motion features present in video stimuli or B) using static photograph stimuli instead of video stimuli so that it was unnecessary to include low-level motion information in our baseline model. We chose to use static image stimuli with a static gabor-wavelet model (capturing orientation and spatial-frequency information)…”

*-The final sentence of introduction seemed to come a bit out of left field?*

We agree, and we have removed this sentence from the introduction. We believe this information makes more sense within the fuller context of the Methods and the Discussion.

*-Methods: bottom of page 25: ‘one of 4 spatial frequencies’, but 5 values listed? Is the ‘0’ spatial frequency no included at each orientation?*

We apologize for the apparent contradiction—yes, that is correct. We have clarified this in the text (p. 31): “Each feature had one of 5 spatial frequencies (0, 2, 4, 8, and 16 cycles per image), and non-zero spatial frequencies had one of 4 orientations (0, 45, 90, or 135 degrees).”

*-Some of the figures in the supplementary material are very hard to read (low res, small). It would be good if any materials that are included are presented at adequate size and resolution.*

We appreciate this feedback. We have now updated the figures to be more legible.

***Have the authors made all data and (if applicable) computational code underlying the findings in their manuscript fully available?*** *The* [*PLOS Data policy*](https://journals.plos.org/ploscompbiol/s/materials-and-software-sharing) *requires authors to make all data and code underlying the findings described in their manuscript fully available without restriction, with rare exception (please refer to the Data Availability Statement in the manuscript PDF file). The data and code should be provided as part of the manuscript or its supporting information, or deposited to a public repository. For example, in addition to summary statistics, the data points behind means, medians and variance measures should be available. If there are restrictions on publicly sharing data or code —e.g. participant privacy or use of data from a third party—those must be specified.*

*Reviewer #1:* ***No:*** *The github link provided is not active so it's not possible to check if the all data aspects are available*

*Reviewer #2:* ***No:*** *I followed the GitHub link but got a 404 error*

Sorry about this. We hadn’t managed to figure out the instructions to make a repository that was selectively accessible to reviewers. The repository should now be public.
